# Evaluation of H_2_ supplementation in *in situ* biomethanation using fed-batch reactors for paper industry wastewater treatment

**DOI:** 10.3389/fmicb.2026.1786024

**Published:** 2026-04-24

**Authors:** Caroline Varella Rodrigues, Maria Leticia Bonatelli, Angel-Maria Thattil, Maria Bernadete Amâncio Varesche, Marcell Nikolausz

**Affiliations:** 1 Biological Process Laboratory, Department of Hydraulics and Sanitation, São Carlos School of Engineering, University of São Paulo (USP), São Carlos, São Paulo, Brazil; 2 Department of Microbial Biotechnology, Helmholtz Centre for Environmental Research (UFZ), Leipzig, Germany

**Keywords:** biomethane, carbon footprint, Ethanoligenenaceae, *Methanobacterium*, Propionibacteriaceae

## Abstract

**Background:**

This study investigated *in situ* biomethanation as a biogas upgrading strategy by injecting hydrogen (H_2_) into anaerobic fed-batch reactors treating wastewater from the pulp and paper industry.

**Methods:**

Granular sludge was used as inoculum and H_2_ was supplied at two pressures (0.6 and 0.9 bar overpressure in CB and CC assays, respectively) to evaluate its impact on treatment efficiency, methane (CH_4_) production, and microbial community dynamics compared to control reactors (CA sets) after an adaptation phase with feeding wastewater only.

**Results:**

The CH_4_ production increased during the first two feeding cycles with H_2_ supplementation accompanied by a reduction in CO_2_ emissions. However, this was transient and at the end of cycle 7 acid accumulation (mainly acetic and propionic acids) and reduced CH_4_ production was observed in both H_2_-supplemented assays. Microbial community structure changed first as a function of new stirred reactor conditions and later according to amount of H_2_ addition resulting in three clearly separated groups of communities. The family Syntrophobacteraceae responsible for propionate degradation declined in all reactors due to operational changes and following microbial succession. In control reactors it was out-competed by members of Geobacteraceae and Desulfobulbaceae. In CB and CC assays, Ethanoligenenaceae, Bacillaceae, Kosmotogaceae, Anaerolineaceae, and Anaerolineaceae families were enriched as a result of H_2_ supplementation. The most abundant methanogens were affiliated to the acetotrophic *Methanothrix* and the hydrogenotrophic *Methanobacterium* in all batch reactors. Upon H_2_ addition the relative abundance of *Methanobacterium* increased and became predominant in later cycles of CB and CC sets. Despite this shift, both genera coexisted throughout the experiments, suggesting that multiple metabolic pathways contributed to CH_4_ production under H_2_-enriched conditions.

**Conclusions:**

Although the process demonstrated potential for simultaneous biogas upgrade and wastewater treatment, the overall performance was negatively influenced by increased H_2_ pressure. This highlights that proper H_2_ dosing and microbial monitoring are critical to ensure process stability for *in situ* biomethanation systems. Considering the fragile balance of the investigated wastewater treatment process an *ex situ* upgrade of biogas using a separate reactor is recommended.

## 1 Introduction

Paper remains an essential commodity in modern life (Liang et al., 2021) and plays a key role in various industrial processes (Jagaba et al., 2022). Global demand for paper goods has been rising, with the market valued at $351.51 billion in 2021, $354.39 billion in 2022, and projected to reach $372.72 billion by 2029 (Kumar et al., 2024). However, the pulp and paper industry has significant environmental impacts, being one of the most polluting and water-intensive sectors. Various processing stages—including cleaning, chemical pulping, bleaching, paper production, equipment cleaning, and cooling—generate large volumes of wastewater, raising serious concerns (Kumar et al., 2024). On average, 50–100 m^3^ of wastewater is produced per ton of pulp and paper (Shabanizadeh and Taghavijeloudar, 2023), with pulp and paper wastewater (PPW) alone accounting for 42% of the 3 billion tons of global industrial wastewater (Liang et al., 2021).

The most concerning compounds in PPW are toxic and include both organic and inorganic materials, such as tannins, resin acids, lignin and its derivatives, extractives, waxes, chlorate ions, dioxins, furans, sulfur compounds, and chemical dyes, as well as adsorbable organic halides (AOX). These pollutants, generated during pulp bleaching with chlorine dioxide, pose serious threats to fish and zooplankton (Ribeiro et al., 2023; Shabanizadeh and Taghavijeloudar, 2023).

PPW have very high chemical oxygen demand (COD), often exceeding 10,000 mg L^−1^, due to substances such as starch, volatile fatty acids (VFAs) from bacterial conversion in recovered paper, and various salts, including calcium carbonate (from pigments), silicates (from deinking and adhesives), and aluminum sulfate (from retention aid additives) (Liang et al., 2021; Shabanizadeh and Taghavijeloudar, 2023; Verma and Chandra, 2024). The wastewater has often a characteristic whitish or brown color, which is primarily caused by lignin and its derivatives severely impacting aquatic ecosystems (Haq and Kalamdhad, 2023).

Given these environmental concerns, proper treatment of PPW is crucial before discharge (Tawfik et al., 2022). Innovative wastewater treatment solutions are needed to eliminate recalcitrant compounds and reduce COD, improving the environmental sustainability of the pulp and paper industry (Ribeiro et al., 2023; Sátiro et al., 2025). With increasing environmental regulations, treating PPW has become economically challenging. However, implementing anaerobic processes such as using anaerobic expanded granular sludge bed (EGSB) or internal circulation (IC) bioreactors show promise, achieving over 70% pollutant removal and offering the added benefit of energy recovery in the form of biogas rich in methane (CH_4_), making it an attractive wastewater treatment option (Liang et al., 2021). However, the direct use of biogas as an energy carrier is limited by the presence of CO₂ (around 30–50%), which must be removed before the upgraded biogas can be injected into the gas grid (Jiang et al., 2021).

Biogas upgrading is a promising method for removing or converting CO_2_ and enhancing the calorific value of biogas (Okoro-Shekwaga et al., 2021; Derakhshesh et al., 2022). Biological upgrade of biogas, also called biomethanation involves the microbial conversion of CO_2_ to CH_4_ by hydrogenotrophic methanogens, using H_2_ as an electron donor. Biological biogas upgrading has advantages such as relatively low investment and operational costs, high efficiency, moderate operating conditions, and high flexibility (Logroño et al., 2021; Hoffstadt et al., 2024). This process recovers energy by producing methane-rich biogas, which meets higher fuel standards and allows for more effective utilization (Okoro-Shekwaga et al., 2021).

Biomethanation can be implemented either *ex situ* or *in situ* depending on the way of H_2_ addition, with the *in situ* process offering advantages such as simpler configuration, greater economic feasibility, and easier operation, making it more attractive. In the case of *in situ* method the H₂ is injected directly into anaerobic digestion (AD) systems used for waste treatment, which can increase CH_4_ content in biogas up to 90% (Wahid et al., 2019; Fu et al., 2021; Derakhshesh et al., 2022). However, there are limitations, particularly in the case of wastewater treatment, such as the potential inhibition of syntrophic oxidation of VFA due to high H_2_ partial pressure negatively influencing the thermodynamic feasibility of the reactions (Luo and Angelidaki, 2013). Nevertheless, numerous successful implementations of *in situ* biomethanation with increased CH_4_ production have been reported using various substrates, including synthetic wastewater (Derakhshesh et al., 2022), food waste (Okoro-Shekwaga et al., 2021), manures and dairy waste, manure co-digested with acidic whey (Luo and Angelidaki, 2013), and pig manure (Zhu et al., 2019).

Although some mechanisms and microorganisms involved in H_2_/CO_2_-to-CH_4_ bioconversion have been identified, the metabolic processes and microbial responses to changes in operating parameters are not fully understood (Fu et al., 2021). According to de Jonge et al. (2022), microbial communities involved in *in situ* biomethanation, which are exposed to changes in operational parameters, remain largely unexplored. The anaerobic microbial system relies on a delicate balance of competing biochemical reactions and syntrophic relationships among microorganisms. Therefore, studying the microbial ecology of H_2_ addition in a balanced wastewater treatment system is crucial. Monitoring changes in community structure will provide insights into optimizing CH_4_ production without compromising wastewater treatment efficiency. Key parameters (e.g., maximum H_2_ conversion rate, impact of H_2_ load on organic matter removal) will inform economic feasibility studies and guide future industrial investments.

This research aimed to study the feasibility of implementation of *in situ* biomethanation into anaerobic treatment of PPW, providing valuable insights into an underexplored area. PPW is often treated by UASB reactors (or similar anaerobic granular sludge reactors such as ESGB or IC reactors) in tall columns, so sparging H_2_ from bottom would mean high hydrostatic pressure. Given these considerations, this study evaluated *in situ* biomethanation in anaerobic fed-batch reactors to assess the impact of H_2_ addition at two different pressures (0.6 and 0.9 bar) on CH_4_ production. The experiments used actual wastewater and granular sludge from a paper company in Saxony, Germany. In addition, this study explored the effect of H_2_ addition on microbial community dynamics through advanced molecular methods.

## 2 Materials and methods

### 2.1 Inoculum and wastewater source

The wastewater and granular sludge mesophilic inoculum were collected from an internal circulation (IC) reactor of a paper company in Trebsen, Germany. The IC reactor treated process wastewater before its subsequent aerobic treatment and proper disposal into the Mulde river. The wastewater from the paper company had a pH of 5.72, a chemical oxygen demand (COD) of 9.04 g L^−1^, and a total ammonia nitrogen concentration of 0.07 g L^−1^. It contained 1,613 mg L^−1^ of lactic acid, 1,960 mg L^−1^ of acetic acid, 110 mg L^−1^ of propionic acid, 524 mg L^−1^ of ethanol, and 173 mg L^−1^ of butyric acid. The wastewater samples were stored in a cold chamber at 4 °C until use.

### 2.2 Experimental setup: fed-batch reactors

**Figure 1:**
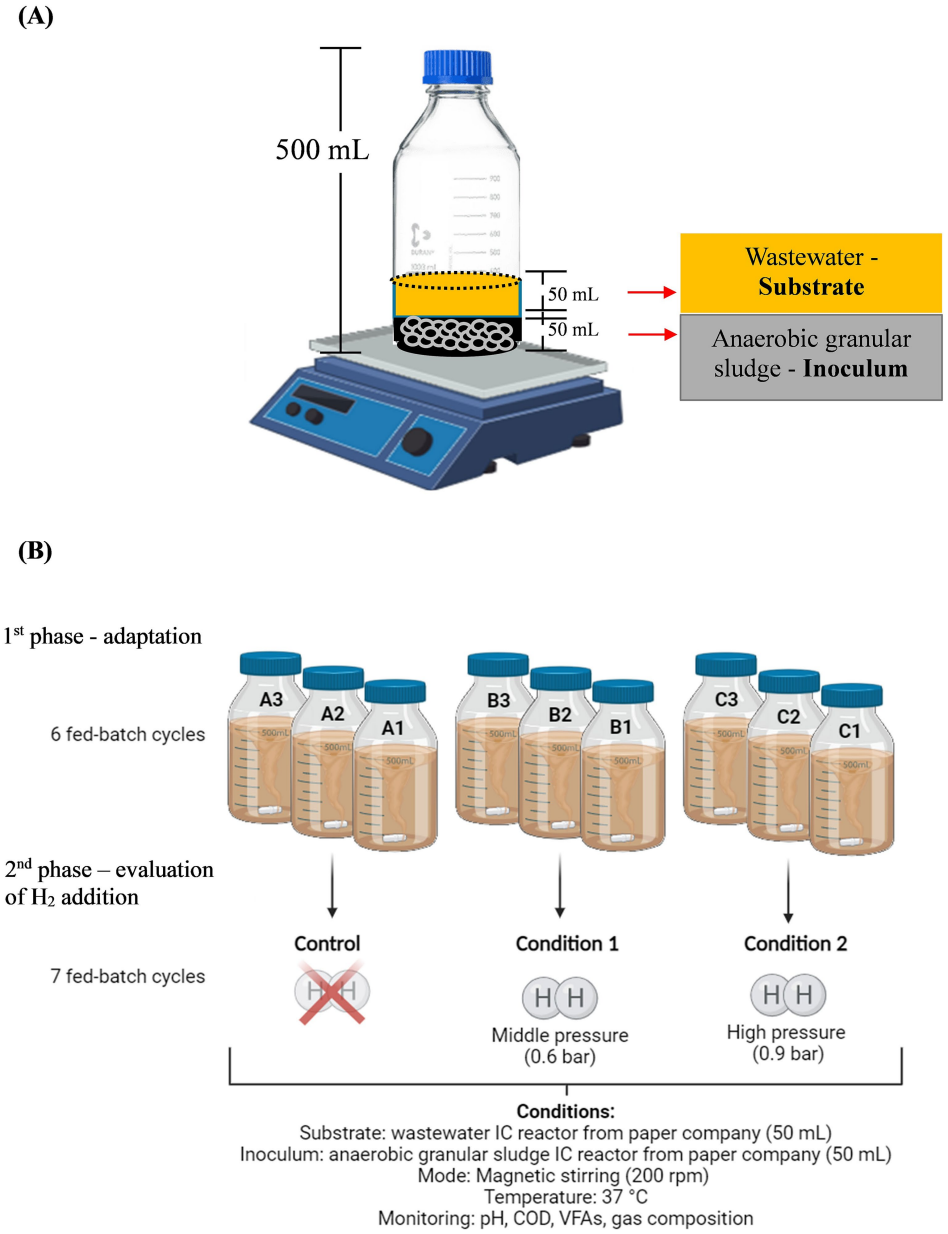
Composition of the anaerobic batch reactors **(A)** and schematic representation of fed-batch reactors **(B)**.

The reactor operation under various conditions was evaluated in two phases: adaptation and evaluation of H_2_ addition. During the adaptation phase (1st phase), seven fed-batch cycles were conducted (three cycles per week, with feeding every 48 h except on weekends) in nine reactors (CA1, CA2, CA3, CB1, CB2, CB3, CC1, CC2, and CC3) to acclimate the inoculum to the substrate and the new reactor conditions. The anaerobic fed-batch reactors (500 mL) were assembled with 50 mL (working volume) of wastewater (substrate) and 50 mL of granular sludge inoculum (Figure 1A). The headspace (400 mL) was flushed with N_2_ (100%) for 5 min, and the reactors were sealed with butyl rubber cork held with plastic cap. Continuous stirring was maintained using magnetic stirrers (200 rpm) at 37 °C. In each fed-batch cycle, the produced gas was released and 50 mL of liquid supernatants were withdrawn before being replaced with an equal volume of fresh wastewater. The hydraulic retention time (HRT) was 2 days, and the organic loading rate (ORL) was 4.52 g COD·L^−1^·d^−1^, calculated based on the influent COD concentration of 9.04 g L^−1^ according to ORL = (COD_influent_ × V_add_ × cycles per day) / V_reactor_.

After the adaptation phase, the reactors proceeded to the evaluation phase, which included the allocation of the reactors into three triplicate sets: a control group (CA1, CA2, and CA3) without H_2_ addition, a group with 0.6 bar overpressure of H_2_ (CB1, CB2, and CB3), and a group with 0.9 bar overpressure of H_2_ (CC1, CC2, and CC3) (Figure 1B). The high pressure value was selected based on a value close to the hydrostatic pressure of 9–10 m liquid column, while 0.6 bar overpressure of H_2_ was considered to convert all produced CO_2_. The same operating conditions and wastewater feeding were maintained during the evaluation step, with seven fed-batch cycles.

### 2.3 Physicochemical and chromatographic analysis

Liquid samples (4 mL) were collected at the beginning and end of each fed-batch cycle and analyzed for pH, COD, and VFAs. Before analysis, the samples were centrifuged (20,817 × g, 4 °C, 10 min), following the procedure described by Logroño et al. (2020), and then filtered through cellulose acetate membrane filters (0.22 μm pore size, 13 mm; LABSOLUTE, Th. Geyer GmbH, Hamburg, Germany). If not analyzed immediately, the samples were stored at −20 °C.

The pH of the liquid samples was measured using a mini pH meter (ISFET pH meter S2K922, ISFETCOM Co., Ltd., Hidaka, Japan). COD was determined using the Hach Lange Cuvette test (LCK014 and LCK1014), while VFAs were analyzed by high-performance liquid chromatography (HPLC) equipped with a refractive index detector (RID) L-2490 and an ICSep COREGEL87H3 column (Transgenomic Inc., Omaha, NE, United States). The sample volume for HPLC analysis was 200 μL, with an injection volume of 20 μL. The HPLC measurements were performed using 5 mM H₂SO₄ as the eluent at a flow rate of 0.7 mL min^−1^.

The relative pressure in the fed-batch reactors was measured using a high-resolution manometer (LEO 5, Keller, Switzerland) connected to a sterile filter (0.2 μm pore size, Ø 25 mm) and a needle, as described by Logroño et al. (2020).

The gas composition in the headspace of the fed-batch reactors was analyzed four times per day, with approximately 2.5-h intervals between each measurement, except on weekends. A gas sample of 1 mL was withdrawn using a syringe and injected into a gas chromatograph (GC). The gas compositions were measured by gas chromatography (InFicon Micro GC Fusion® Gas Analyzer, INFICON, Bad Ragaz, Switzerland) equipped with a thermal conductivity detector with the columns Rt-Molsieve and Rt-Q-Bond with the column temperatures 80 °C and 60 °C, respectively.

### 2.4 Microbial community analysis

Liquid samples (1.0 mL) were collected at the start and at the end of the 3rd, 5th, 6th, and 7th cycles of the anaerobic fed-batch reactor operation. The pellets obtained after centrifugation (4 °C, 20,817 × g, 5 min) were used to isolate genomic DNA using the NucleoSpin Soil kit (MACHEREY-NAGEL GmbH & Co., KG, Germany) with buffer SL2, following the manufacturer’s protocol. The extracted DNA was stored at −20 °C until use.

The microbial community composition was analyzed by amplicon sequencing of the *mcrA* genes for methanogens and the 16S rRNA genes for Bacteria. The V3–V4 region of the 16S rRNA genes was amplified using primers 341f and 785r (Klindworth et al., 2013). For methanogens, the mlas and mcrA-rev primers were used (Steinberg and Regan, 2008). Amplicon sequencing was performed on the Illumina MiSeq platform using the MiSeq Reagent Kit v3 with 2 × 300 cycles, according to Logroño et al. (2020). Sequence analyses were performed by applying default parameters unless specified otherwise. Demultiplexed paired-end reads were processed with DADA2 version 1.24.0 software package (Callahan et al., 2016) on R software environment (Vienna, 2022) using cutadapt version 4.0 (Martin, 2011) to remove primer sequences from reads. The parameters used for 16S RNA gene sequences filtering and trimming were [truncLen = c(260,240), maxN = 0, maxEE = c(2, 2), truncQ = 2], while for mcrA genes the following parameters were used [truncLen = c(260,230), maxN = 0, maxEE = c(8, 8), truncQ = 2]. Sample inference was made considering the learned error rate, and then the paired-end reads were merged. Chimeras were removed considering the method consensus. The Silva database (version 138.1) (Quast et al., 2013) was used for taxonomy assignment of 16S RNA genes, while a custom taxonomy database of *mcrA* sequences was used as described by Logroño et al. (2020) for the taxonomic affiliation of the methanogens. The sequences were rarefied to the lowest read numbers of the samples (16S rRNA gene sequence libraries: 25808 sequences; mcrA gene sequence libraries: 16781 sequences). Spearman’s correlation analysis was done with R stats package (R Core Team, 2024), after the normalization of the amplicon data with centered log-ratio function from R compositions package (van den Boogaart et al., 2025). The sequencing data are deposited in the NCBI public database under the BioProject accession number PRJNA1374211.

### 2.5 Biogas production

The volume of gasses consumed and produced during the anaerobic reactor operation was calculated using equation based on Avogadro’s law (Equation 1).


P.V=n.R.T (1)

Where:

*P* = pressure (bar).

*V* = headspace volume in the Glass Bottle in Liter (L).

*n* = gas produced in moles (mol).

*T* = temperature in Kelvin (K).

*R* = gas constant (0.0831446261815324 L·bar·K^−1^·mol^−1^).

## 3 Results

### 3.1 Evaluation of the pH and COD in the fed-batch assays

**Figure 2:**
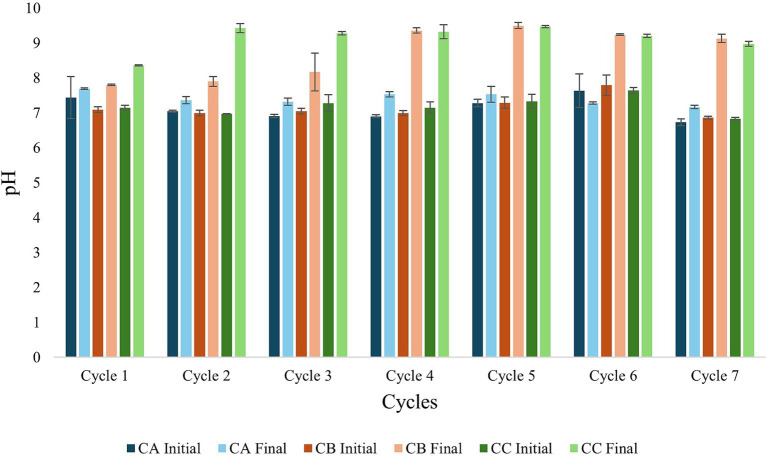
Evaluation of pH during cycles 1–7 in anaerobic fed-batch reactors during the experimental phase of hydrogen addition to CB and CC reactors.

**Figure 3:**
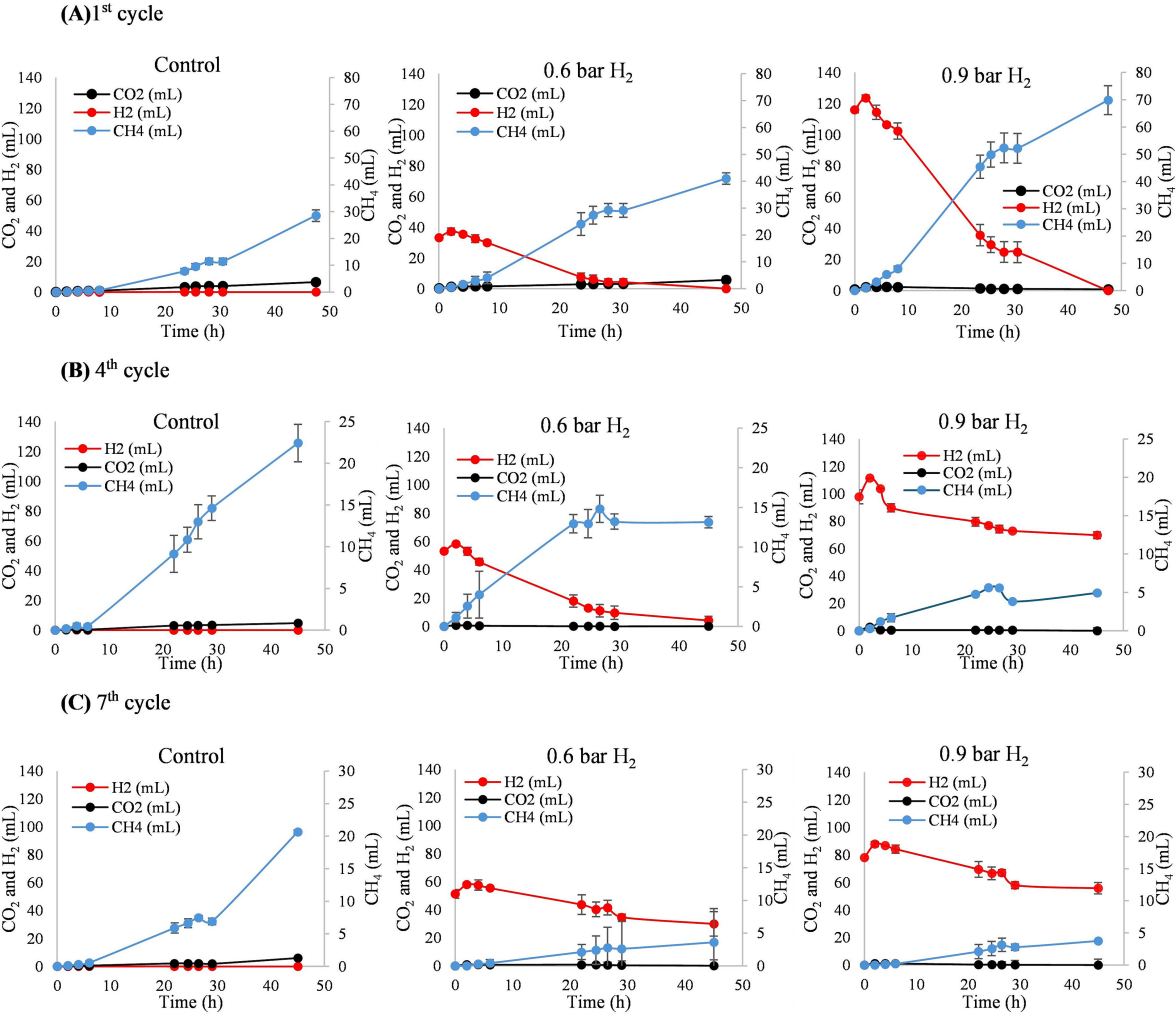
Evaluation of CO_2_, H_2_, and CH_4_ during the 1st cycle **(A)**, 4th cycle **(B)**, and 7th cycle **(C)**.

The final pH values of the anaerobic fed-batch reactors were higher than the initial values across all experimental cycles (Figure 2). This increase was particularly pronounced in assays CB and CC and coincided with a progressive depletion of CO_2_ (Figure 3).

A paired *t*-test revealed significant differences in pH between CA and CB (*t* = −4.38, *p* = 0.0047) and between CA and CC (*t* = −9.58, *p* < 0.0001). These results indicate that the increase in pH observed in the control assay (CA) was significantly lower than that observed in the H_2_-amended assays (CB and CC). Notably, although an increase in pH was also detected in assay CA, the magnitude of this change remained consistently lower than that observed in CB and CC throughout the operational cycles.

### 3.2 Effect of hydrogen addition on the removal efficiency of VFAs

**Figure 4:**
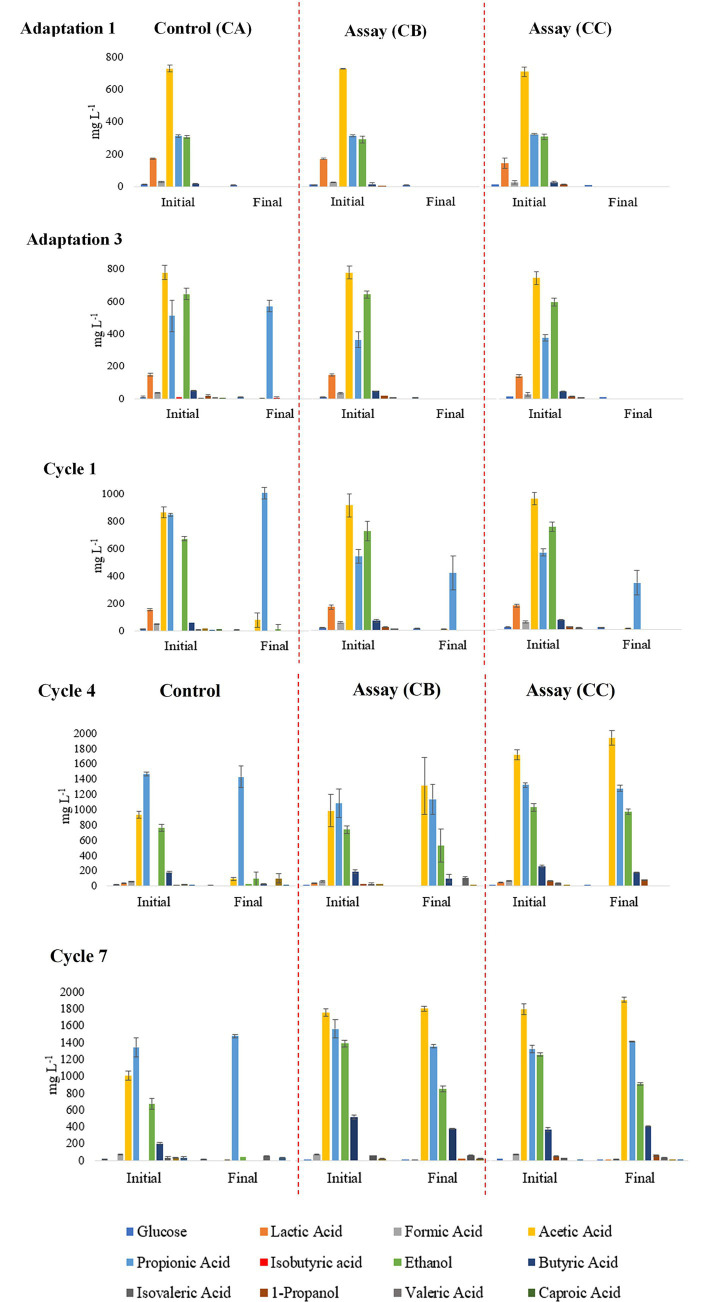
Evaluation of VFAs, glucose, and alcohol concentrations at the initial (after feeding of wastewater) and final phases of a feed cycle (before starting the new feeding) in fed-batch reactors at selected cycles of adaptation and experimental period with H_2_-addition.

Accumulation of propionic acid was observed in all assays, particularly at the end of the second adaptation phase in the control assay (CA) and at the end of Cycle 1 in the assays with H_2_ addition (CB and CC) (Figure 4). In addition, lactic acid was detected at the beginning of the operational cycles, from Adaptation 1 to Cycle 3 (Supplementary Figure C).

### 3.3 H_2_ use efficiency and CH_4_ generation

At 0.6 bar overpressure, during the 1st and 4th cycles during the experimental period, CH_4_ production was directly proportional to H_2_ consumption. However, in the 5th and 7th cycles, the system became saturated, and the added H_2_ was not fully consumed, which were also observed by the limited CH_4_ production. Factors such as the reactor’s operational mode and the accumulation of propionic acid may have contributed to this reduction in methane generation. At 0.9 bar of H_2_, a decline in both methane production and H_2_ consumption was already evident from the 4th cycle onward, indicating that *in situ* biomethanation becomes inefficient under elevated H_2_ pressures in a fed-batch system.

### 3.4 Bacteria

#### 3.4.1 Beta diversity analysis based on 16S rRNA gene amplicon sequencing

**Figure 5:**
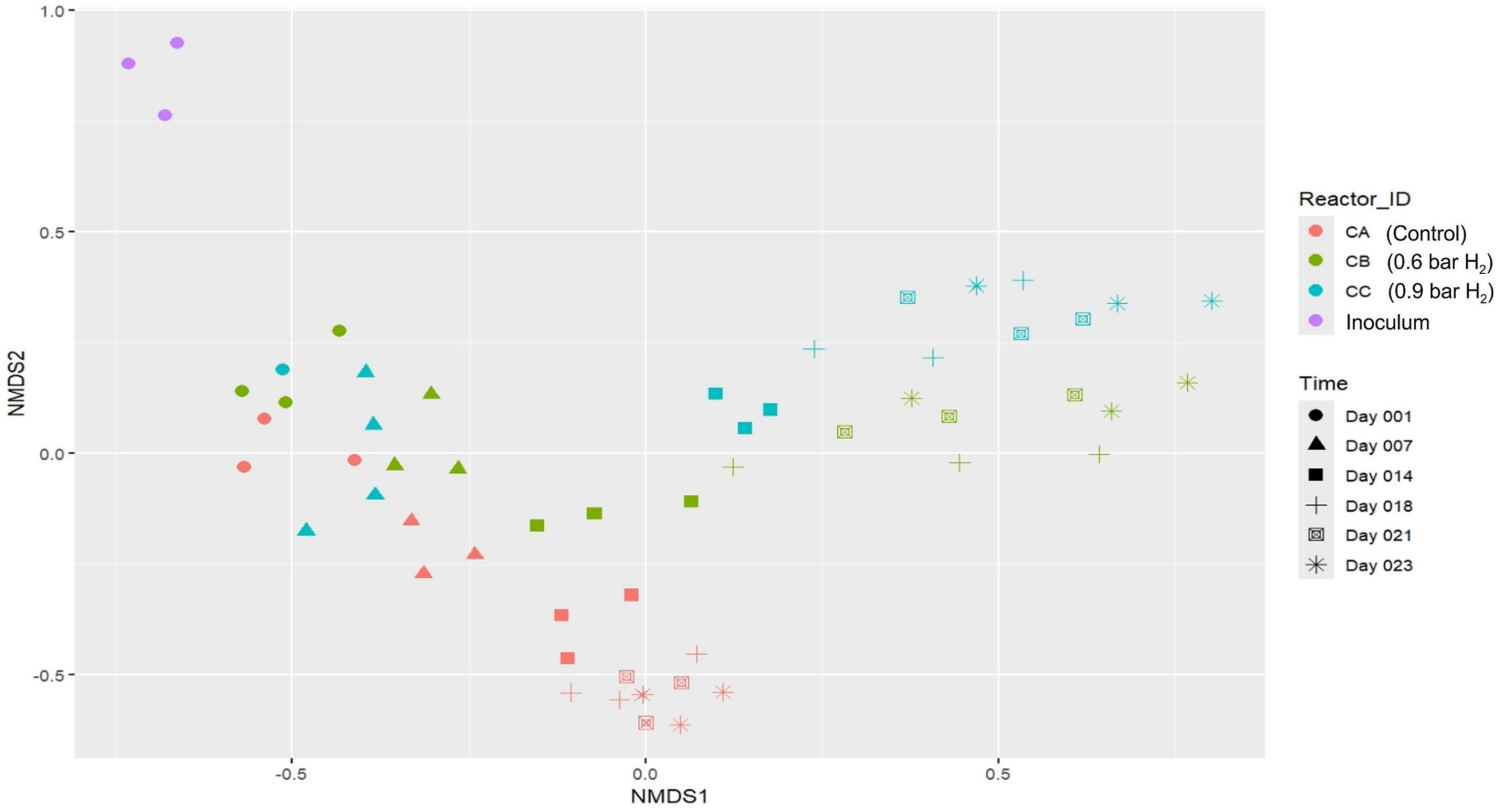
Beta-diversity visualized using non-metric multidimensional scaling (NMDS) plot with Bray–Curtis dissimilarity distances. NMDS plot based on community structures of bacteria described by amplicon sequencing of the 16S rRNA gene. Assay CA: control; CB: 0.6 bar H_2_; CC: 0.9 bar H_2_. Sampling points: Day 1 (Adaptation 1), Day 7 (Adaptation 3), Day 14 (Cycle 3), Day 18 (Cycle 5), Day 21 (Cycle 6), and Day 23 (Cycle 7). Stress = 0.1714048.

Non-metric multidimensional scaling (NMDS) based on 16S rRNA gene sequencing data was performed to assess temporal dynamics and treatment-induced changes in the bacterial community structures among the fed-batch reactors at various phases. As shown in Figure 5, samples from the inoculum clustered separately from those collected during reactor operation, indicating a distinct initial microbial profile prior to exposure to experimental conditions.

Throughout the experimental period, samples from the CA reactors (control, without H_2_ supplementation) followed a clear successional trend across the NMDS space, reflecting a gradual and consistent shift in the bacterial community over time. In contrast, reactors CB and CC, which received H_2_ at partial pressures of 0.6 and 0.9 bar, respectively, exhibited more marked divergence in their microbial community structures, particularly from Day 014 (Cycle 3) onwards. These observations suggested that H_2_ supplementation imposed a strong selective pressure, driving the formation of distinct microbial assemblages relative to the control.

A clear separation of the CB and CC samples became evident after Day 018 (Cycle 5), indicating distinct microbial assemblages developing under hydrogen supplementation. This divergence contrasts with the gradual and consistent successional trend observed in the control reactor (CA), emphasizing the influence of increasing H_2_ partial pressure on bacterial community differentiation under *in situ* biomethanation conditions.

#### 3.4.2 Community structure of the Bacteria

The most abundant phyla in both the inoculum and all three types of reactors were Actinobacteriota, Bacteroidota, Chloroflexi, Firmicutes, Proteobacteria, Verrucomicrobiota, Nitrospirota. Further changes were observed as a function of H_2_ addition within the phyla Thermotagae, Synergistota.

**Figure 6:**
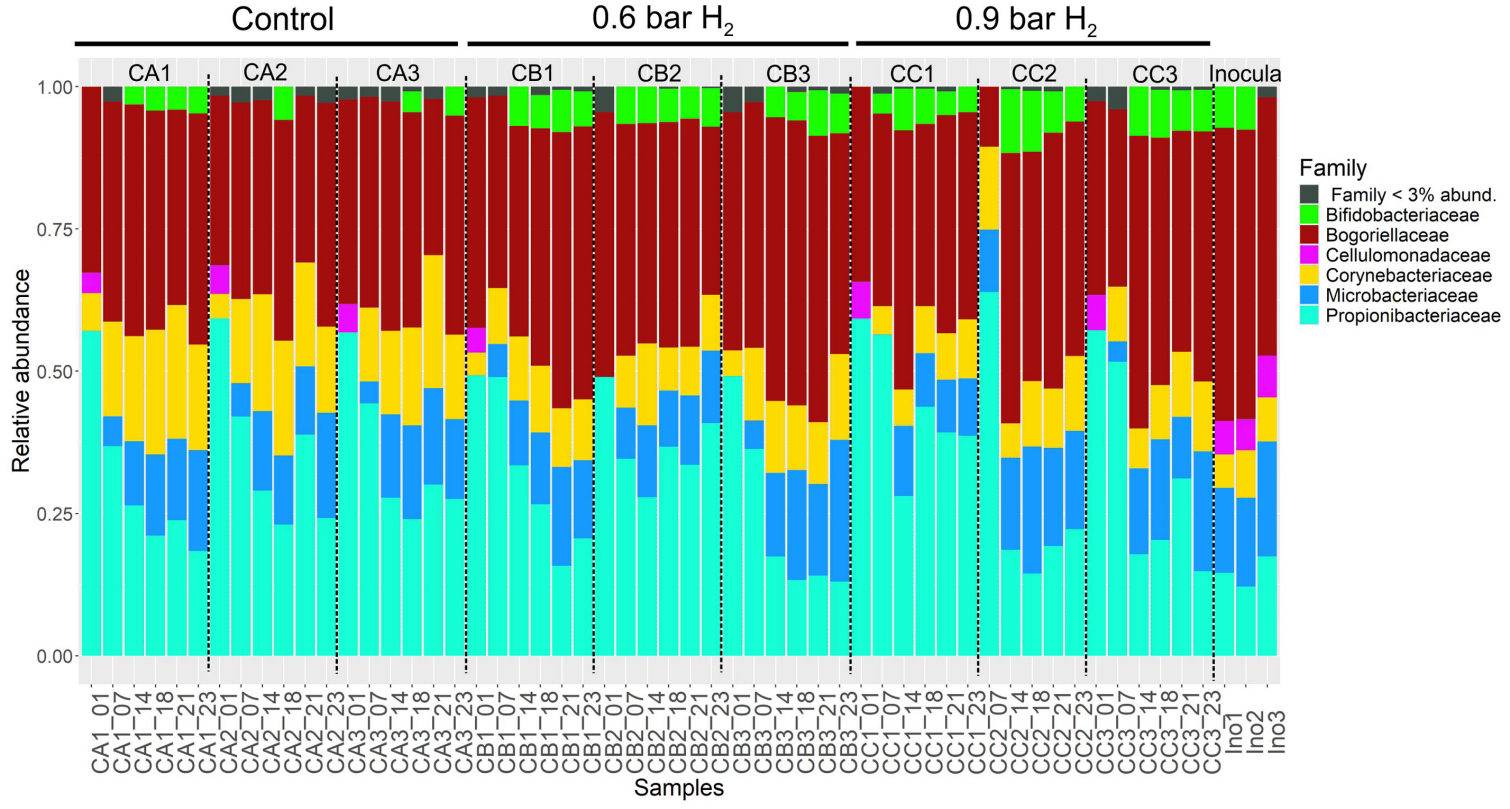
Composition and relative abundance of families within the *Actinobacteriota* phylum across different anaerobic fed-batch reactor conditions derived from the rarefied dataset. Stacked bar plots show the distribution and temporal variation of Actinobacteriota families in samples from control (CA), 0.6 bar H_2_-supplemented (CB), and 0.9 bar H_2_-supplemented (CC) reactors, along with the initial inoculum (Ino). Day 1 (Adaptation 1), Day 7 (Adaptation 3), Day 14 (Cycle 3), Day 18 (Cycle 5), Day 21 (Cycle 6), and Day 23 (Cycle 7).

The abundance of Actinobacteriota families were generally lower in early stages of the experiment, with a progressive increase over time in the hydrogen-amended treatments (Figure 6). Especially the abundance of families Propionibacteriaceae and Bogoriellaceae increased in comparison to the control reactors (Figure 6). This indicates that members of these families are more competitive under increased hydrogen partial pressures.

The Kosmotogaceae family, classified within the phylum Thermotogae, was the dominant group across all assays. Its relative abundance was notably higher in assay CB compared to assays CA and CC (Supplementary Figure D).

Members of the Synergistaceae family (phylum Synergistota) were detected at high relative abundance across the assays (Supplementary Figure E), with particularly elevated levels observed in assay CA, which did not receive H_2_ supplementation. Acetoclastic methanogens, notably *Methanotrix*, were also detected at high relative abundance in this assay.

Elevated concentrations of propionic acid were detected in all assays, with the highest values observed in assay CC, which received high H_2_ addition. In this assay, propionate concentrations ranged from approximately 747 to 1,539 mg L^−1^ (Figure 4). At the end of the final cycles in assays CB and CC, the pH of the medium increased to 9.31 and 9.28, respectively (Figure 2).

Microorganisms belonging to the Lentimicrobiaceae family (phylum Bacteroidota) were detected at high relative abundance in assay CA (control, without H_2_ addition). Their abundance showed an increasing trend over successive fed-batch cycles (Supplementary Figure F). In contrast, in the assays supplemented with H_2_ (CB and CC), members of this family were not detected after H_2_ addition, although they were observed at low relative abundance during the initial adaptation phase across all assays.

Members of the Dysgonomonadaceae family were consistently detected across all assays throughout the experimental cycles. Elevated concentrations of volatile fatty acids, including propionic acid, were observed under alkaline conditions, with pH values close to 9 maintained during the operation.

The Anaerolineaceae family was detected at high relative abundance across all assays (Supplementary Figure G), indicating its consistent presence throughout the anaerobic digestion process.

**Figure 7:**
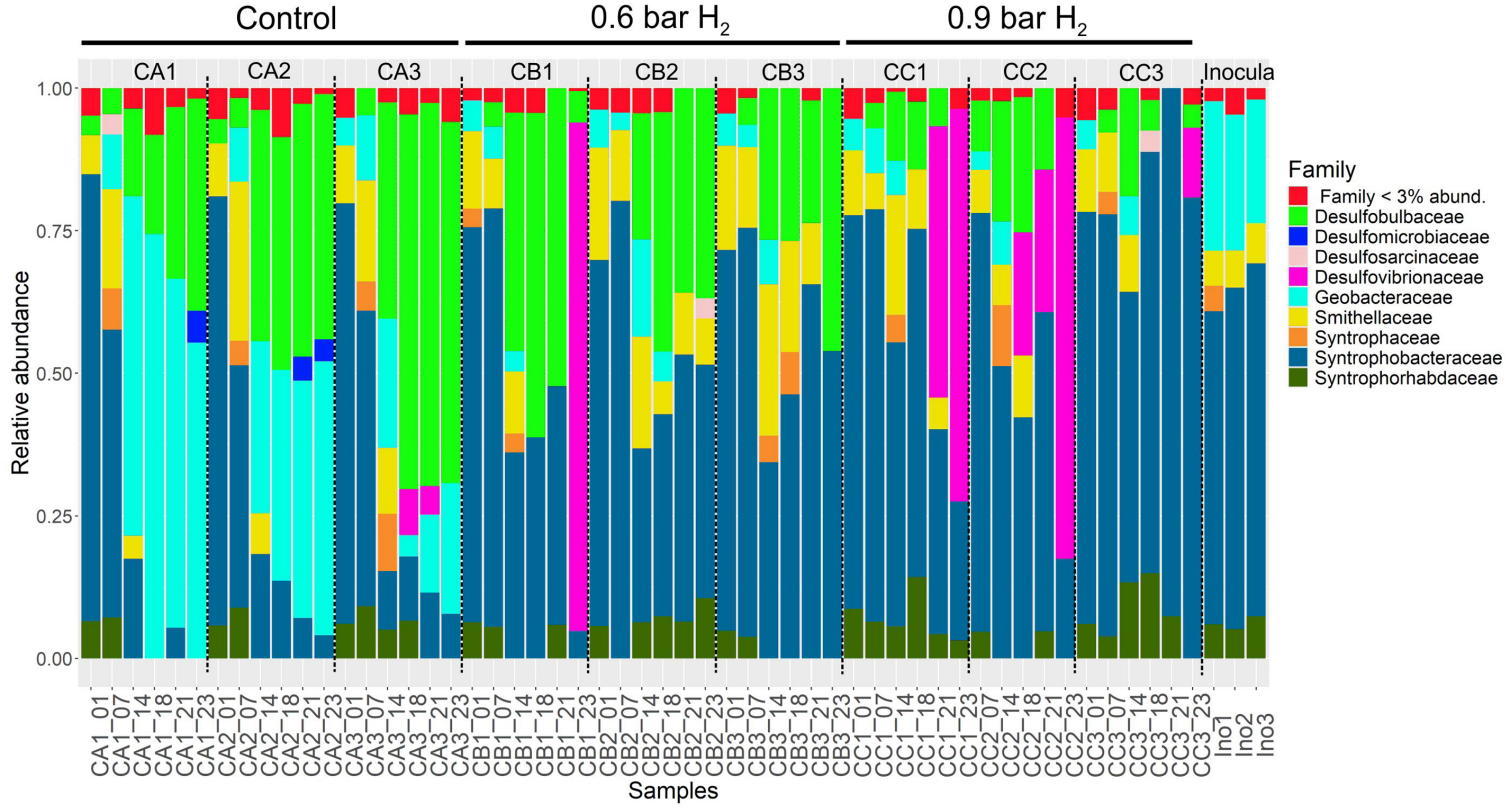
Composition and relative abundance of Desulfobacterota families across anaerobic fed-batch reactor samples derived from the rarefied dataset. Stacked bar charts show the distribution of Desulfobacterota families among the control (CA), 0.6 bar H_2_-supplemented (CB), and 0.9 bar H_2_-supplemented (CC) samples, as well as the initial inoculum (Ino). Day 1 (Adaptation 1), Day 7 (Adaptation 3), Day 14 (Cycle 3), Day 18 (Cycle 5), Day 21 (Cycle 6), and Day 23 (Cycle 7).

The most pronounced reduction in propionic acid was observed up to Adaptation 3, followed by a less marked decrease during Cycle 1 in assays CA, CB, and CC. From Cycle 3 onward, a gradual decline in the relative abundance of Syntrophobacteraceae was observed across all assays (CA, CB, and CC) (Figure 7). This decrease coincided with the persistence of propionic acid at the end of the experimental period.

An increased relative abundance of Ethanoligenenaceae was observed in the assays with H_2_ supplementation (CB and CC) (Supplementary Figure J). Higher ethanol concentrations were also detected in these reactors compared to the control assay.

### 3.5 Methanogens

#### 3.5.1 Beta diversity analyses of methanogens based on *mcrA* gene amplicon sequences

**Figure 8:**
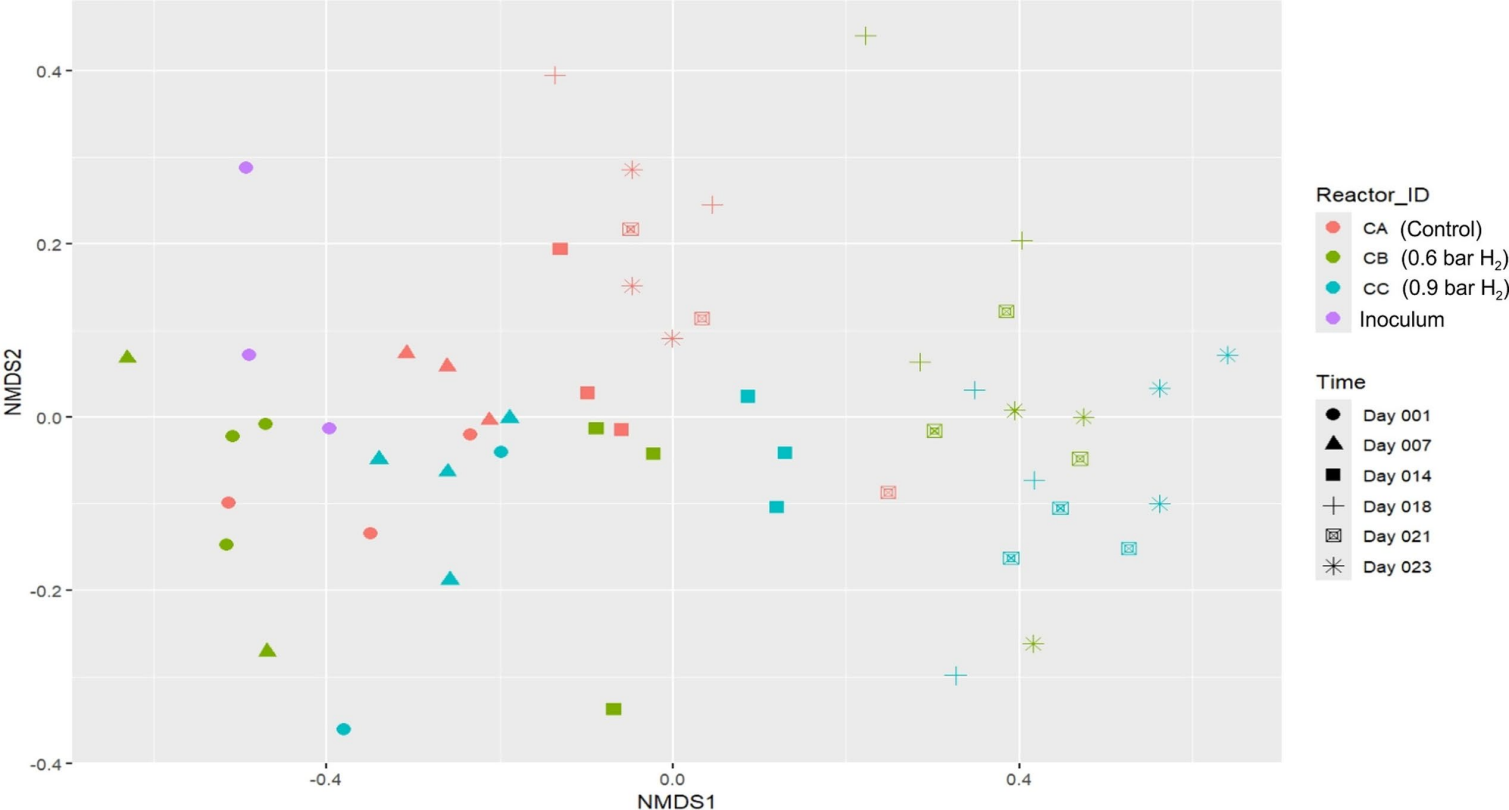
Beta-diversity visualized using non-metric multidimensional scaling (NMDS) plot with Bray–Curtis dissimilarity distances. Methanogenic community structures were assessed by using amplicon sequencing of the *mcrA* gene. Assay CA: control; CB: 0.6 bar H_2_; CC: 0.9 bar H_2_. Sampling points: Day 1 (Adaptation 1), Day 7 (Adaptation 3), Day 14 (Cycle 3), Day 18 (Cycle 5), Day 21 (Cycle 6), and Day 23 (Cycle 7). Stress = 0.1464375.

To investigate the shifts in methanogenic community structures throughout the fed-batch experiments, NMDS analysis was also conducted based on *mcrA* gene sequencing. As illustrated in Figure 8, the methanogenic archaea communities in the initial inoculum were clearly separated from those in the reactor samples, reflecting community restructuring in response to the changes of reactor conditions from a large-scale IC reactor setup with continuous feeding to a stirred reactor setup with fed-batch mode, as well as reflecting effect of external H_2_ addition.

#### 3.5.2 Community structures of the methanogens

**Figure 9:**
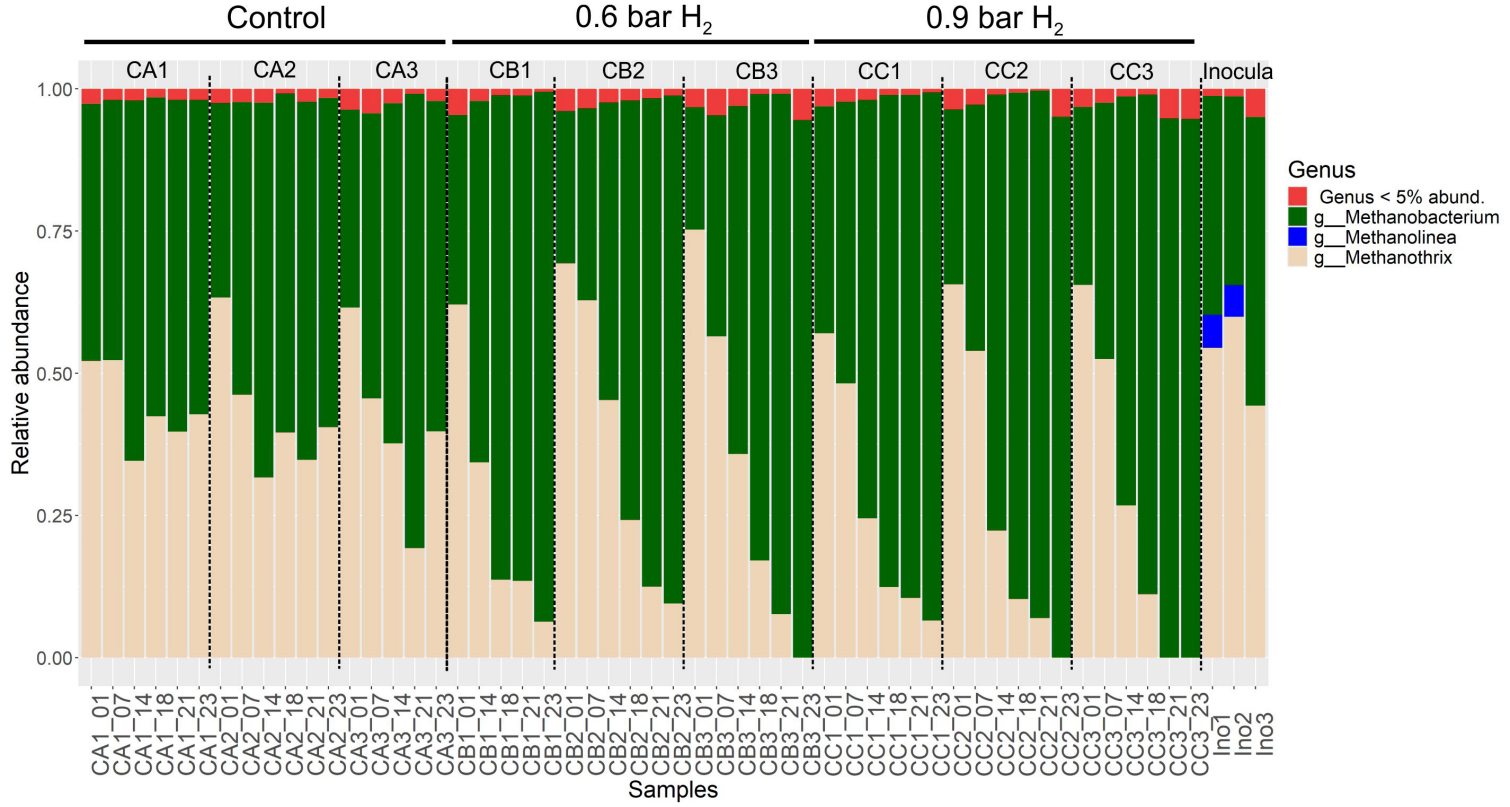
Composition and relative abundance of methanogenic archaeal genera in anaerobic fed-batch reactors under different hydrogen partial pressures. Stacked bar plots display the abundance of methanogenic genera (based on the *mcrA* gene) in samples from control (CA), 0.6 bar H_2_-supplemented (CB), and 0.9 bar H_2_-supplemented (CC) anaerobic reactors, as well as the original inoculum (Ino). The abundance values were derived from rarefied data set. Samples were collected on Day 1 (Adaptation 1), Day 7 (Adaptation 3), Day 14 (Cycle 3), Day 18 (Cycle 5), Day 21 (Cycle 6), and Day 23 (Cycle 7).

The methanogenic archaeal community was initially dominated by *Methanothrix* in the inoculum, and this profile remained predominant in the control assay (CA), which operated without external H_2_ addition (Figure 9). In contrast, reactors supplemented with H_2_ (CB and CC) exhibited a marked shift in community composition, characterized by a decline in *Methanothrix* and an increase in the relative abundance of *Methanobacterium*. During the final days of operation in assays CB and CC, *Methanobacterium* reached its highest relative abundance, concomitant with a decrease in *Methanothrix*.

## 4 Discussion

### 4.1 Evaluation of the pH and COD in the fed-batch assays

The increase in pH observed in assays CB and CC can be attributed to CO_2_ consumption during hydrogenotrophic methanogenesis, which involves the conversion of CO_2_ and H_2_ into CH_4_ (Equation 2). As CO_2_ is highly soluble in aqueous systems, bicarbonate remaining in the liquid phase may have been utilized by hydrogenotrophic microorganisms in the presence of H_2_, resulting in CH_4_ formation and a concomitant rise in pH (Agneessens et al., 2017).


$$4H_{2}+CO_{2}\to CH_{4}+2H_{2}O\;\;\;\;$$ (2)

The removal of bicarbonate likely reduced the buffering capacity of the system, leading to a marked pH increase, particularly between the second and seventh operational cycles (Figure 2). In addition, the consumption of volatile fatty acids (VFAs), which are primarily responsible for lower pH values in the initial samples, further contributed to the pH elevation observed in certain samples.

Although pH also increased in the control assay, this effect was less pronounced and may be attributed to identical operational conditions applied across reactors. Specifically, periodic degassing and refilling of the headspace with N_2_ during each cycle may have promoted CO_2_ desorption from the liquid phase, gradually shifting the bicarbonate equilibrium and leading to pH increases, as previously reported by Agneessens et al. (2017).

Luo and Angelidaki (2013) reported that highly alkaline pH values can inhibit methanogenic activity. In this study, during cycles 5 to 7, CO_2_ depletion may have caused substrate limitation for hydrogenotrophic methanogenic microorganisms, which rely on CO_2_ as a carbon source. Kwang et al. (2023) reported an increase in pH to ≥8.0 was directly associated with a decline in the CH_4_ production rate. The resulting pH increase could have hindered CH_4_ production, as observed in this study (Figure 3).

Szuhaj et al. (2016) observed that a reduction in CO_2_ was accompanied by an increase in pH, as CO_2_ is highly soluble in the aqueous medium and can be removed from the system either through a methanogenic reaction with H_2_ (Equation 2) or by degassing the reactors. The authors reported a pH increase from 8.66 to 9.38 following *in situ* biomethanation due to CO_2_ depletion. Similarly, Oliveira et al. (2024) attributed the rise in pH to the consumption of dissolved inorganic carbon (DIC) by hydrogenotrophic methanogens. Luo and Angelidaki (2013) found that the addition of H_2_ to a continuously stirred tank reactor led to a pH increase, which significantly impacted the growth rates of acidogens and methanogens.

According to the data presented in the Supplementary Figure B, a reduction in organic matter was observed up to cycle 5, which is expected in anaerobic digestion processes. This reduction in COD indicates that part of the available carbon was converted into methane (gas phase), while another fraction was transformed into secondary metabolites. In cycles 6 and 7, however, particularly in the CC assay of cycle 6 and the CB and CC assays of cycle 7, the absence of COD reduction may be positively associated with a decreased efficiency in organic matter removal, likely related to the increasing H_2_ feeding applied to the batch anaerobic reactors.

### 4.2 Effect of hydrogen addition on the removal efficiency of VFAs

Several studies have demonstrated that specific bacterial species, such as *Propionibacterium freudenreichii*, are capable of utilizing lactic acid as a carbon source and converting it into propionic acid in repeated batch fermentations (Bezirci et al., 2023). This metabolic feature has been exploited as a strategy to enhance propionic acid production in fed-batch systems through the conversion of lactic acid (Sabra et al., 2013).

In the present study, the lactic acid detected during the initial operational phases originated from the paper industry wastewater (from Adaptation 1 to Cycle 3—Supplementary Figure C) and may have served as a substrate for propionate-producing bacteria, contributing to the observed increase in propionic acid concentrations. This effect may have been further intensified by the fed-batch operating mode, which has been reported to favor propionate accumulation by promoting repeated substrate availability and selective enrichment of propionate-producing microorganisms (Sabra et al., 2013).

In addition, the accumulation of propionic acid can also be attributed to the high partial pressure of H_2_ during the later cycles of the experimental period. Rocamora et al. (2023) reported that the inhibition of propionic acid degradation could be positively correlated with an H_2_ partial pressure exceeding 10^−4^ bar. Tsapekos et al. (2022) also observed that an increase in H_2_ partial pressure led to a higher concentration of propionic acid, as similarly observed in the present study.

According to Guo et al. (2021), propionic acid degradation can occur through two pathways via syntrophic propionate-oxidizing bacteria (SPOB): the methylmalonyl coenzyme A randomization pathway (Equation 3) and the dismutation pathway (Equation 4). In both cases, the products generated (acetate, formate, and H_2_) must be efficiently removed to thermodynamically favor propionate degradation. However, both pathways are thermodynamically inhibited by high H_2_ concentrations (Guo et al., 2021). Therefore, in the present study, the degradation of propionic acid appears to have been thermodynamically hindered due to the high H_2_ partial pressures observed in the CB and CC assays.


$$\begin{array}{l} C_{2}H_{5}\mathrm{CO}O^{-}+3H_{2}O\to CH_{3}\mathrm{CO}O^{-}(\text{acetate})\\ +\mathrm{HC}O_{3}^{-}+H^{+}+3H_{2} \end{array}$$ (3)

$$2C_{2}H_{5}\mathrm{CO}O^{-}+2H_{2}O\to 3CH_{3}\mathrm{CO}O^{-}(\text{acetate})+H^{+}+2H_{2}$$ (4)

According to Jønson et al. (2022), propionic acid accumulation can be considered as an indicator of stress in anaerobic digestion systems. Guo et al. (2021) reported that inefficient *ex situ* methanogenesis leads to propionic acid accumulation due to residual H_2_. Consistent with Guo et al. (2021), the present study observed that following H₂ addition, the propionic acid concentration increased from 373 to 1,491 mg L^−1^ at 0.6 bar H_2_ and from 302 to 1,539 mg L^−1^ at 0.9 bar H_2_, coinciding with residual H_2_ in both assays.

Capson-Tojo et al. (2017) observed that when the operating time of batch reactors is not long enough, propionic acid can accumulate. Therefore, the accumulation of this acid observed at the end of all assays in the present study could be positively correlated with the operational mode. In fed-batch mode, the feeding intervals were short (approximately 48 h), which was not sufficient for the reduction of propionic acid.

According to Agneessens et al. (2017), the H_2_ injected into the reactors can be consumed by both hydrogenotrophic methanogenic archaea and homoacetogenic bacteria, as outlined in Equations 5 and 6, respectively (Rachbauer et al., 2017).


$$4\;H_{2}+CO_{2}\to CH_{4}+2H_{2}O(\Delta G{}^{\circ}\prime \;=\;-135.6\;\mathrm{kJ/mol})$$ (5)

$$4\;H_{2}+2CO_{2}\to CH_{3}\text{COOH}+2H_{2}O(\Delta G{}^{\circ}\prime \;=\;-94.9\;\mathrm{kJ/mol})$$ (6)

Homoacetogenic bacteria can consume up to 40% of the H_2_ added to the reactors, as the half-velocity constant (Ks) for these microorganisms in converting H_2_ to acetic acid is approximately 10 times higher than that of hydrogenotrophic methanogens (Agneessens et al., 2017). Lecker et al. (2017) and Akimoto et al. (2024) reported that the activity of homoacetogenic bacteria was positively correlated with higher H_2_ partial pressure compared to hydrogenotrophic methanogenic archaea. Rachbauer et al. (2016) observed homoacetogenic activity during the initial phase of the operation of a trickle-bed reactor, when hydrogenotrophic methanogen biomass was still growing, and the partial pressure of H_2_ remained high.

In addition to the possibility that the accumulation of acetic acid observed at the end of the assays with H_2_ addition (from Cycle 2 in assay CC and from Cycle 3 in assay CB) was related to the activity of homoacetogenic bacteria, it is important to highlight that this accumulation may also be associated with the decrease in the acetoclastic methanogenic community and the inhibition of syntrophic acetate oxidizing bacteria (SAOB), which would otherwise consume this acid. The supplementation with H_2_ may have suppressed acetoclastic methanogens and SAOB, thereby creating favorable conditions for the growth of hydrogenotrophic methanogenic archaea.

### 4.3 H_2_ use efficiency and CH_4_ generation

The observed reduction in methane generation under elevated H_2_ pressures may be related to the operational characteristics of the fed-batch reactors and to the accumulation of inhibitory intermediates, particularly propionic acid. In assays CB and CC, propionate accumulation played a critical role in limiting CH_4_ production.

The conversion of propionic acid to acetic acid is thermodynamically unfavorable, and its accumulation can suppress the activity of acetoclastic methanogens, thereby impairing biogas production (Han et al., 2020; Zhu et al., 2023). Once accumulated, propionic acid disrupts anaerobic digestion by inhibiting acetate conversion to methane, ultimately reducing overall methanogenic performance (Ping et al., 2022).

Together, these findings suggest that, under fed-batch conditions, elevated H_2_ partial pressures may promote metabolic bottlenecks associated with propionate accumulation, limiting both H_2_ utilization and methane production during *in situ* biomethanation.

### 4.4 Bacteria

#### 4.4.1 Community structure of the bacteria

Members of the Kosmotogaceae family are known to engage in syntrophic interactions with hydrogenotrophic methanogens, including *Methanoculleus* and *Methanobacterium* (Rhee et al., 2021). Therefore, the prevalence of Kosmotogaceae observed in this study may be positively associated with syntrophic relationships involving these archaeal groups, potentially contributing to enhanced CH_4_ generation under H_2_-enriched conditions.

The Synergistaceae family comprises fermentative bacteria capable of producing acetic and propionic acids and is known for establishing syntrophic interactions with acetoclastic methanogenic archaea (Wang et al., 2024; Baena et al., 1998; Chertkov et al., 2010). The high relative abundance of Synergistaceae observed in assay CA supports their role in acid production and suggests a syntrophic relationship with acetoclastic methanogens, particularly *Methanotrix*. This association may have contributed to maintaining acetate turnover and methanogenic activity under conditions without H_2_ supplementation.

Members of the Propionibacteriaceae family (phylum Actinobacteriota) are well known for their ability to produce propionic acid, typically using glucose or lactic acid as carbon sources and converting them into propionate, as reported in batch fermentation systems (Ng et al., 2023; de Assis et al., 2022). The elevated propionate concentrations observed in the present study, particularly under high H_2_ conditions in assay CC, may be associated with the activity of microorganisms from this family. Additionally, Propionibacteriaceae have been reported to contribute substantially to volatile fatty acid production under alkaline conditions, particularly at pH values around 9 (Chen et al., 2022). This is consistent with the pH values measured at the end of the final cycles in assays CB and CC, suggesting that alkaline conditions may have favored acid production activity in these reactors.

Members of the Lentimicrobiaceae family are commonly associated with anaerobic digestion systems enriched in starch-rich substrates (Sun et al., 2016). These strict anaerobes are known to degrade carbohydrates and produce metabolites such as acetate, malate, formate, and hydrogen (Alves et al., 2022). The detection of Lentimicrobiaceae during the adaptation phase in all assays is likely linked to the starch content of the paper-processing wastewater used in this study, as previously reported for anaerobic environments with high-strength starch (Aghasa et al., 2022). The persistence and increasing abundance of this microbial group in assay CA suggest that the fed-batch operational mode favored their maintenance under control conditions. Conversely, the absence of Lentimicrobiaceae following H_2_ addition indicates a potential inhibitory effect of elevated H_2_ partial pressure in assays CB and CC.

Dysgonomonadaceae family comprise hydrolytic, non-spore-forming bacteria capable of degrading recalcitrant polysaccharides into oligosaccharides and monosaccharides and have been strongly associated with volatile fatty acid (VFA) production in anaerobic digesters (Shamurad et al., 2020). Previous studies have reported significant positive correlations between Dysgonomonadaceae abundance and propionic, butyric, and acetic acid concentrations, as well as total VFA levels (Owusu-Agyeman et al., 2022). In particular, Owusu-Agyeman et al. (2022) demonstrated that alkaline conditions (pH 9) favor the predominance of this family and are linked to increased propionic acid production. In the present study, the consistent detection of Dysgonomonadaceae across all assays suggests their involvement in the degradation of polysaccharides derived from paper and pulp wastewater. Their persistence under pH values close to 9 indicates that alkaline conditions may have supported their metabolic activity and contribution to acid production.

Anaerolineaceae have been widely reported as key contributors to the hydrolytic and acidogenic stages of anaerobic digestion, participating in the breakdown of carbohydrates and other complex organic compounds (Hamze et al., 2024). Members of this family are fermentative bacteria capable of producing volatile fatty acids (e.g. acetic acid), H_2_, and CO_2_, thereby supporting both acetoclastic and hydrogenotrophic methanogenic archaea (McIlroy et al., 2017). In addition, syntrophic interactions between Anaerolineaceae and methanogens such as *Methanobacterium* and *Methanothrix* have been previously described, mediated by hydrogen and/or formate transfer (Ude et al., 2024). The high relative abundance of Anaerolineaceae observed across all assays in the present study suggests that this family may have played an important role in sustaining syntrophic networks essential for methane production.

Members of the Syntrophobacteraceae family function as syntrophic propionate-oxidizing bacteria (SPOB) and are capable of utilizing propionate as an electron donor during sulfate reduction (Liu and Conrad, 2017). The higher relative abundance of this family observed during the initial stages of the experiments may be positively associated with propionate removal. Conversely, the decline of Syntrophobacteraceae from Cycle 3 onward suggests a reduced capacity for propionate oxidation, which may explain the accumulation of propionic acid toward the end of the assays. This reduction in activity and growth may have been influenced by changes in the operational conditions of the fed-batch reactors. Similar trends were reported by Giordani et al. (2019), who observed a decrease in Syntrophobacter populations under a 24-h cycle time.

In the assays with H_2_ addition (CB and CC), the excess hydrogen likely inhibited the syntrophic oxidation of propionate, since this process, carried out by Syntrophobacteraceae, is thermodynamically unfavorable and requires extremely low partial pressures of H₂ (Jin and Lu, 2023). In the control assays (CA), the reduction in Syntrophobacteraceae abundance may be attributed to the natural succession of the microbial community over the operational cycles, which favored the proliferation of microorganisms from the Geobacteraceae family (Figure 7), whose relative abundance increased throughout the operation. Members of the Geobacteraceae family are known to play a crucial role in the degradation of organic acids and in direct electron transfer to methanogenic archaea, particularly in environments with limited availability of intermediate electron acceptors such as H_2_ or formate (Wang et al., 2021). This aligns with the detection of these microorganisms in the control assays (CA) of the present study. Consequently, Geobacteraceae may have outcompeted Syntrophobacteraceae in occupying ecological niches, especially under the more stable environmental conditions observed during fed-batch operation.

Moreover, the potential for direct interaction between Geobacteraceae and acetoclastic archaea such as *Methanothrix* through direct interspecies electron transfer (Wu et al., 2022) may confer advantages by reducing reliance on classical hydrogen-dependent syntrophic pathways, such as those mediated by Syntrophobacteraceae. These findings suggest that, although Syntrophobacteraceae are metabolically capable of degrading propionate, unfavorable factors such as elevated H_2_ partial pressure and specific operational conditions in fed-batch systems may limit their persistence and functional activity over time, as observed in the present study.

Members of the Ethanoligenenaceae family are known to persist under alkaline conditions and primarily produce ethanol and organic acids, while exhibiting a comparatively limited capacity for hydrogen production (Diez et al., 2024). The enrichment of this family in the H_2_-supplemented assays may, therefore, be associated with the elevated pH values characteristic of these reactors. In addition, the concurrent increase in ethanol concentrations suggests a positive relationship between Ethanoligenenaceae abundance and ethanol production. Previous studies have shown that the abundance of this family is strongly influenced by pH (Penna et al., 2025), supporting the hypothesis that higher pH conditions favored their proliferation in assays CB and CC.

### 4.5 Methanogens

#### 4.5.1 Beta diversity analyses of methanogens based on *mcrA* gene amplicon sequences

The CA reactor, which operated without H_2_ supplementation, displayed a progressive but moderate change in community structure over time, remaining relatively similar to the inoculum compared to the hydrogen-amended treatments. Conversely, reactors CB and CC, subjected to *in situ* biomethanation with H_2_ pressures of 0.6 and 0.9 bar, respectively, exhibited substantial divergence from both the inoculum and the control reactor samples. This divergence became more pronounced as the operation progressed, suggesting that hydrogen partial pressure strongly influenced the selection and enrichment of specific methanogenic populations.

In contrast to the clearer clustering observed in the 16S rRNA gene profiles, the NMDS ordination of the metagenomic data did not reveal a strong separation between the two H_2_-amended setups (CB and CC). Although some directional shifts along the NMDS1 axis are visible, particularly in later sampling days, the overall trajectories of CB and CC remain partially overlapping. This suggests that, under the tested conditions, elevated H_2_ availability induced compositional changes but not a distinctly divergent restructuring of the methanogenic community between the application of two different H_2_ partial pressures. The reactors were not feed with hydrogen over the weekend, which might have some minor effect on the microbial communities. According to our previous study (Logroño et al., 2021) the microbial communities are functionally resilient upon starvation of H_2_ + CO_2_ feeding and even longer-term of starvation (7 to 14 days) had negligible effect on the community structure.

#### 4.5.2 Community structures of the methanogens

*Methanothrix* is an obligate acetoclastic methanogen that relies exclusively on acetate as its substrate, and its high abundance in the inoculum and in assay CA is consistent with acetate consumption for CH_4_ production (Hellal et al., 2024). This interpretation is supported by the rapid acetate removal observed in assay CA (Figure 4, Cycle 7). The introduction of H_2_ promoted a shift toward hydrogenotrophic methanogenesis, favoring the dominance of *Methanobacterium*. This adaptive response under *in situ* biomethanation conditions agrees with previous findings reporting a negative correlation between *Methanothrix* abundance and H_2_ availability (Braga-Nan et al., 2025). Accordingly, the reduced relative abundance of *Methanothrix* observed in assays CB and CC suggests that elevated H_2_ availability altered substrate utilization patterns and selectively favored hydrogenotrophic methanogens, mainly *Methanobacterium*, over acetoclastic methanogens.

#### 4.5.3 Correlation analyses

**Figure 10:**
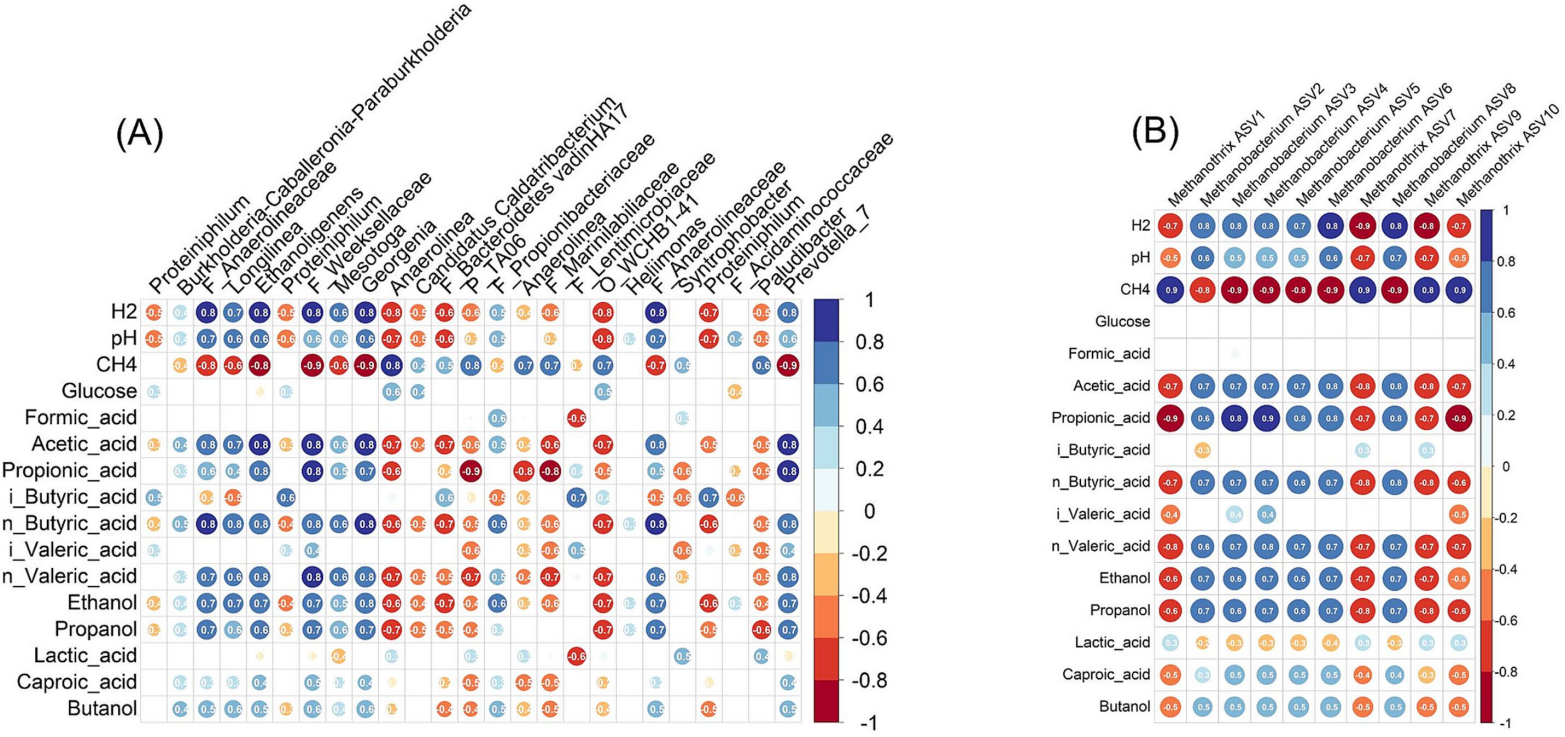
Spearman’s correlation between bacterial **(A)** and methanogenic archaea **(B)** community members and batch bioreactor operating parameters. The heatmap shows Spearman’s correlation coefficients between the normalized counts of dominant taxa (rows) and key reactor parameters (hydrogen, pH) and waste conversion products (glucose, organic acids, alcohols, methane). Only the top 25 amplicon sequence variants (ASVs) representing ~50% of bacteria abundance **(A)** and top 10 ASVs of methanogenic archaea represents ~70% of the abundance **(B)** were used in the analysis. We are showing the genera to which the ASVs belong, in case higher taxonomic levels are shown, there are depicted with capital letters (P – phylum, O – order, F – family). For archaea (B), ASV numbers are shown. Blue and red colors indicate positive and negative correlations, respectively, with color intensity proportional to the correlation magnitude. Only statistically significant values (*p* < 0.05) are shown.

Figure 10 shows the correlation analyses between the process parameters and the top ASVs for both bacterial and methanogenic archaea community members (Figure 10). The major parameter we changed in our experiment was the supplementation of different amounts of H_2_, and strongest positive correlation was found between H_2_ and the genera *Longilinea*, *Ethanoligens*, *Georgenia*, *Prevotella* and taxa (not affiliated at genus level) belonging to families Anaerolineaceae and Weeksellaceae. Those taxa also presented positive correlation to many of the conversion products, such as organic acids and alcohol (Figure 10A). Interestingly, the genus *Anaerolinea* showed negative correlation with H_2_, while related taxa at family level (Anaerolineaceaea) positively correlated with it. On the other hand, genera *Proteiniphilum*, *Candidatus* Caldatribacterium, and families Marinilabiliacea, Bacteroidetes vadinHA17, which presented a negative correlation with H_2_, also showed negative correlation to many of the conversion products. This suggest that those taxa were negatively influenced by the H_2_ addition (Figure 10A).

The trend in case of methanogens was clearer. The most abundant amplicon sequence variants (ASVs) affiliated to the strict hydrogenotrophic *Methanobacterium* genus were positively correlated with H_2_, while those affiliated with the strict acetotrophic *Methanothrix* were negatively correlated with it. Interestingly, acetic acid as a substrate for acetotrophic methanogenesis negatively correlated with *Methanothrix*. This can be explained by the inhibition and decrease of this genus, which resulted in reduced consumption and; therefore, an accumulation of acetic acid. Similar correlation was observed with the other accumulating alcohols and organic acids (Figure 10B).

## 5 Conclusion

This study demonstrated the limitations of *in situ* biomethanation as a strategy to enhance CH_4_ production from paper industry wastewater through the controlled addition of H_2_ in anaerobic fed-batch reactors. Among the tested conditions, a medium H_2_ pressure (0.6 bar) during the first two feeding cycles notably but only transiently improved CH_4_ production (47.5 and 13.14 mL, respectively), while also contributing to a reduction in CO_2_ emissions. The observed increase in final pH across all assays was attributed to the depletion of CO_2_ by hydrogenotrophic methanogens and reactor headspace degassing following each feeding cycle. Microbial community analysis revealed a gradual shift from an initial dominance of strict acetoclastic methanogens (*Methanothrix*) toward hydrogenotrophic methanogens (*Methanobacterium*), driven by repeated H_2_ supplementation.

Overall, the findings indicate that *in situ* biomethanation is not a suitable strategy for the paper industry context, as it can compromise process stability and wastewater treatment performance. Considering that biogas production represents only a minor by-product rather than a main economic goal for these facilities, *ex situ* biogas upgrading might be considered as a more robust and controllable alternative not directly influencing the wastewater treatment efficiency. Although *ex situ* biomethanation would require the establishment of an additional reactor with the associated costs, the process could be independently operated and optimized (e.g., thermophilic process could be implemented with the associated higher specific methane productivity). This study therefore provides valuable insights into microbial interactions under H_2_-enriched conditions and supports the design of more sustainable and efficient bioenergy recovery strategies for industrial wastewater management.

## Data Availability

The datasets presented in this study can be found in online repositories. The names of the repository/repositories and accession number(s) can be found in the article/Supplementary material.
