# Supplementary material for: Evaluation of H2 supplementation in in situ biomethanation using fed-batch reactors for paper industry wastewater treatment
Source: Front Microbiol. 2026 Apr 24;17:1786024. doi: 10.3389/fmicb.2026.1786024 (PMC13153053; doi:10.3389/fmicb.2026.1786024)
Supplement: Supplementary file 1 [file Table_1.DOCX]

**Supplementary Material**

**Evaluation of H₂ supplementation in *in situ* biomethanation using fed-batch reactors for paper industry wastewater treatment**

Caroline Varella Rodrigues^a,b*^, Maria Leticia Bonatelli^b^, Angel-Maria Thattil^b^, Maria Bernadete Amâncio Varesche^a^, Marcell Nikolausz^b^

^a^Department of Hydraulics and Sanitation, São Carlos School of Engineering, University of São Paulo (USP). 1100 João Dagnone Avenue, São Carlos, SP, 13563120, Brazil.

^b^Department of Microbial Biotechnology, Helmholtz Centre for Environmental Research -UFZ, Leipzig, Germany.

**Figure A**: Evaluation of CO_2_, H_2_ and CH_4_ during the 5^th^ cycle.

**Figure B**: Evaluation of COD over the cycles during the fed-batch reactors operation.

**Figure C:** Evaluation of VFAs, glucose, and alcohol concentrations at the initial and final phases of fed-batch reactor operation for each adaptation and cycle.


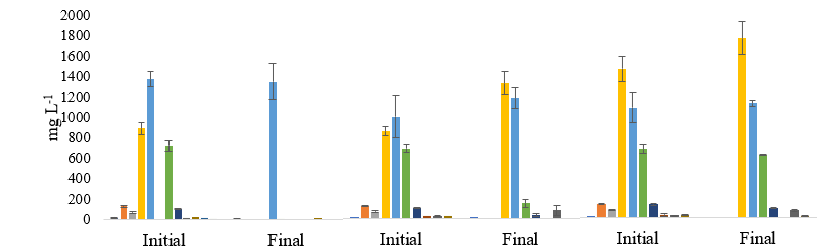

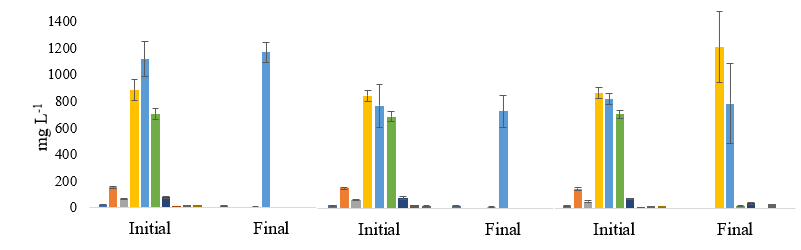


**Control (CA)**

**Assay (CB)**

**Assay (CC)**


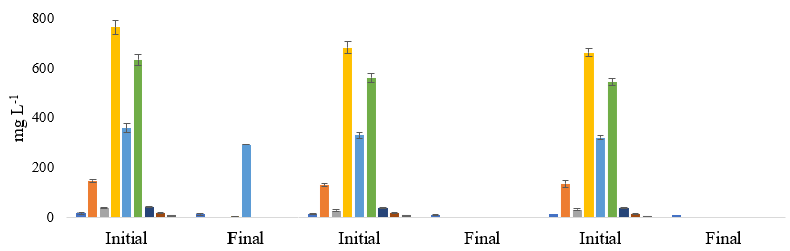


**Adaptation 2**

**Cycle 2**

**Cycle 3**


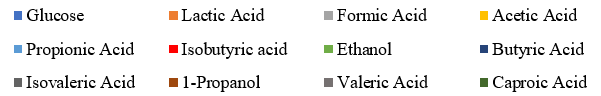


**Cycle 6**

**Control (CA)**

**Assay (CB)**

**Assay (CC)**


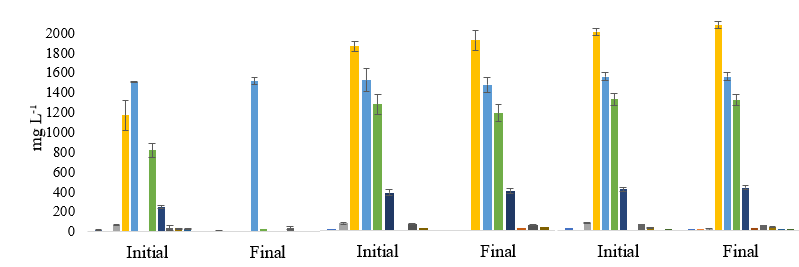


**Cycle 5**


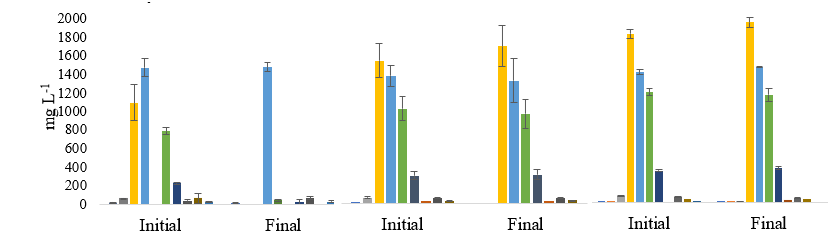


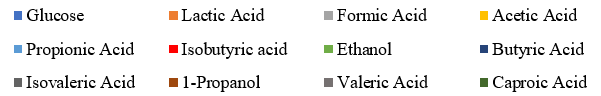


**Figure D**: Relative abundance of *Thermotogota* families over time in fed-batch reactor samples.

**CA1**

**CA2**

**CA3**

**CB1**

**CB2**

**CB3**

**CC1**

**CC2**

**CC3**

**Inoc**

**Abundance**

**Family**

**Kosmotogaceae**


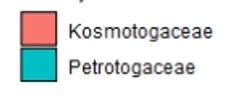


**Petrotogaceae**

**Sample**


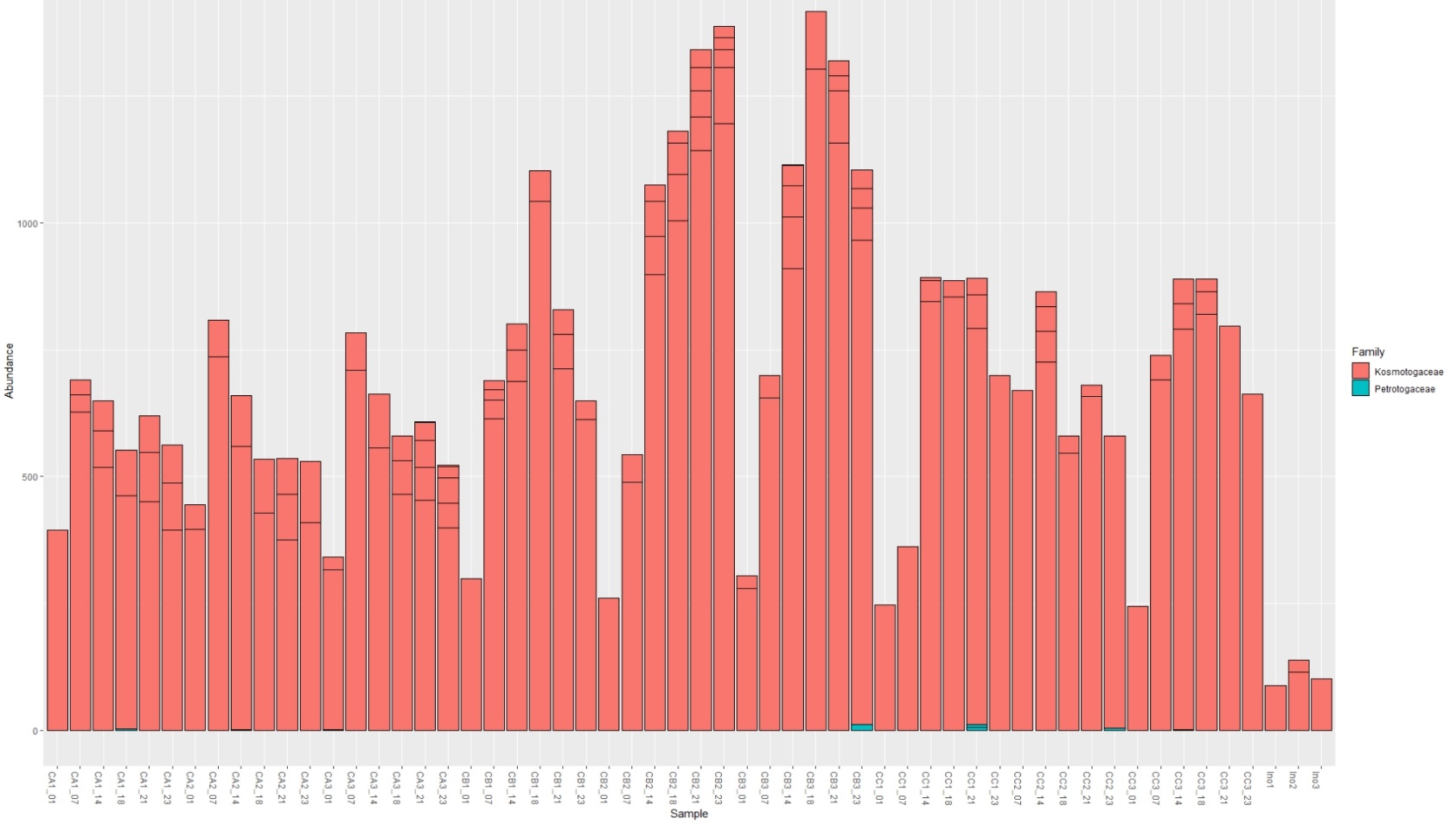


The bar plot shows the abundance of the families Kosmotogaceae (pink) and Petrotogaceae (blue) across samples from reactors CA (control), CB (0.6 bar H₂), and CC (0.9 bar H₂), as well as from the initial inoculum (Inoc), throughout the experimental period. Kosmotogaceae was predominant in all samples, while Petrotogaceae appeared at low abundance and only sporadically, particularly in reactors CB and CC from Day 014 (Cycle 3) onward. These results indicate a consistent presence of Kosmotogaceae regardless of H_2_, suggesting its resilience under different operational conditions. Assay CA: control (CA1–CA3 = triplicates 1–3); Assay CB: 0.6 bar H₂ (CB1–CB3 = triplicates 1–3); Assay CC: 0.9 bar H₂ (CC1–CC3 = triplicates 1–3). Day 1 (Adaptation 1), Day 7 (Adaptation 3), Day 14 (Cycle 3), Day 18 (Cycle 5), Day 21 (Cycle 6), and Day 23 (Cycle 7).

**Figure E:** Temporal dynamics of the *Synergistaceae* family across fed-batch reactor samples.


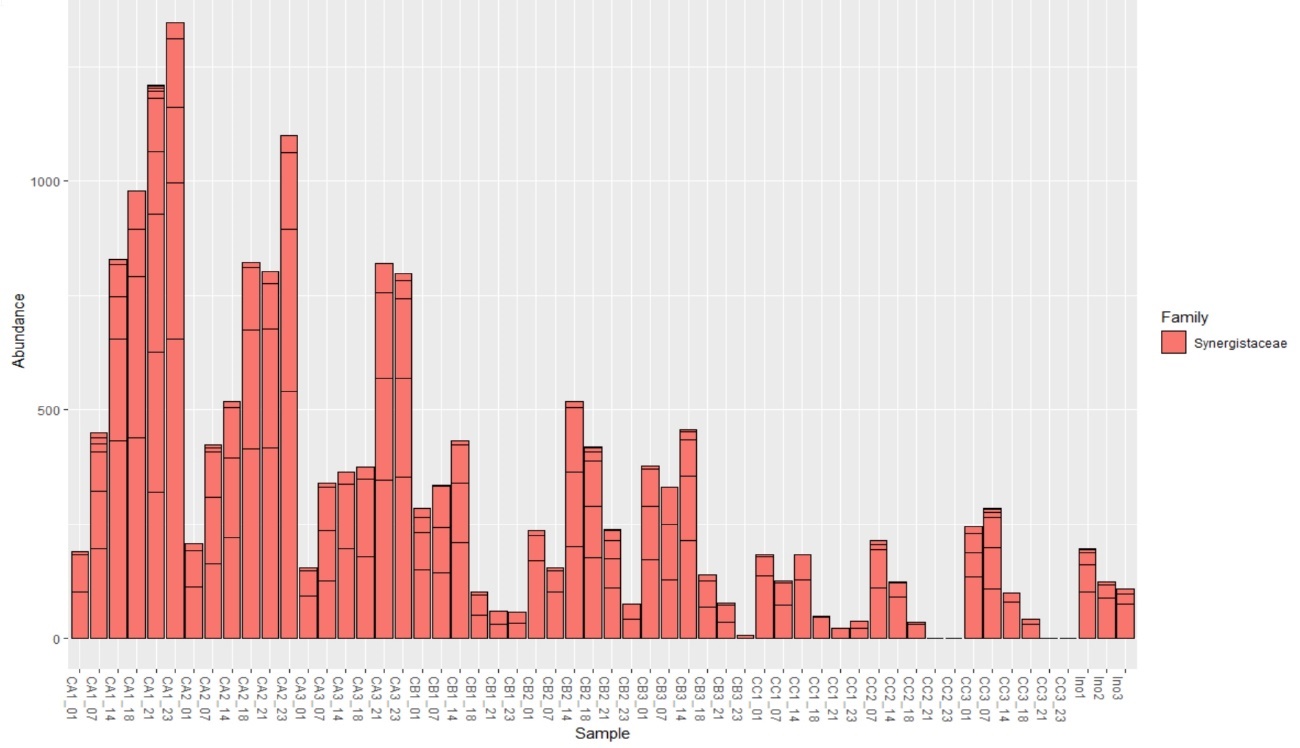


**CA1**

**CA2**

**CA3**

**CB1**

**CB2**

**CB3**

**CC1**

**CC2**

**CC3**

**Inoc**

**Abundance**

**Family**

**Synergistaceae**


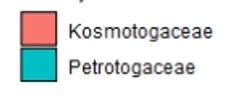


**Sample**

The bar plot presents the abundance of Synergistaceae in samples collected from the control reactor (CA), and reactors supplemented with H_2_ at 0.6 bar (CB) and 0.9 bar (CC), along with the initial inoculum (Ino). A clear decrease in Synergistaceae abundance over time is observed in all treatments, particularly in hydrogen-supplemented reactors. This suggests a potential sensitivity of this family to increased hydrogen partial pressures, with stronger suppression at higher doses (notably in reactor CC). The trend highlights the selective pressure of H₂ on fermentative microbial populations within the Synergistota phylum. Assay CA: control (CA1–CA3 = triplicates 1–3); Assay CB: 0.6 bar H₂ (CB1–CB3 = triplicates 1–3); Assay CC: 0.9 bar H₂ (CC1–CC3 = triplicates 1–3). Day 1 (Adaptation 1), Day 7 (Adaptation 3), Day 14 (Cycle 3), Day 18 (Cycle 5), Day 21 (Cycle 6), and Day 23 (Cycle 7).

**Figure F:** Distribution of families within the *Bacteroidota* phylum across anaerobic fed-batch reactor samples.


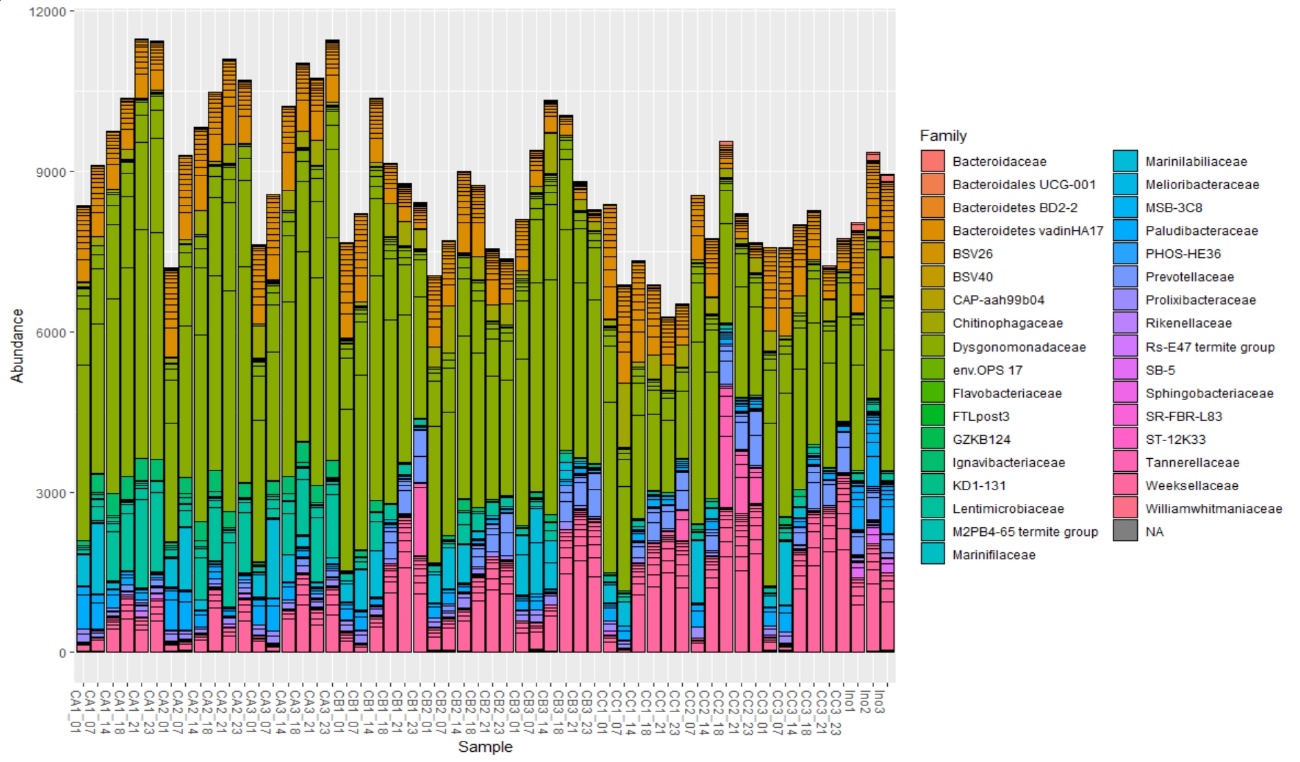


**CA1**

**CA2**

**CA3**

**CB1**

**CB2**

**CB3**

**CC1**

**CC2**

**CC3**

**Inoc**

**Abundance**

**Family**

**Sample**


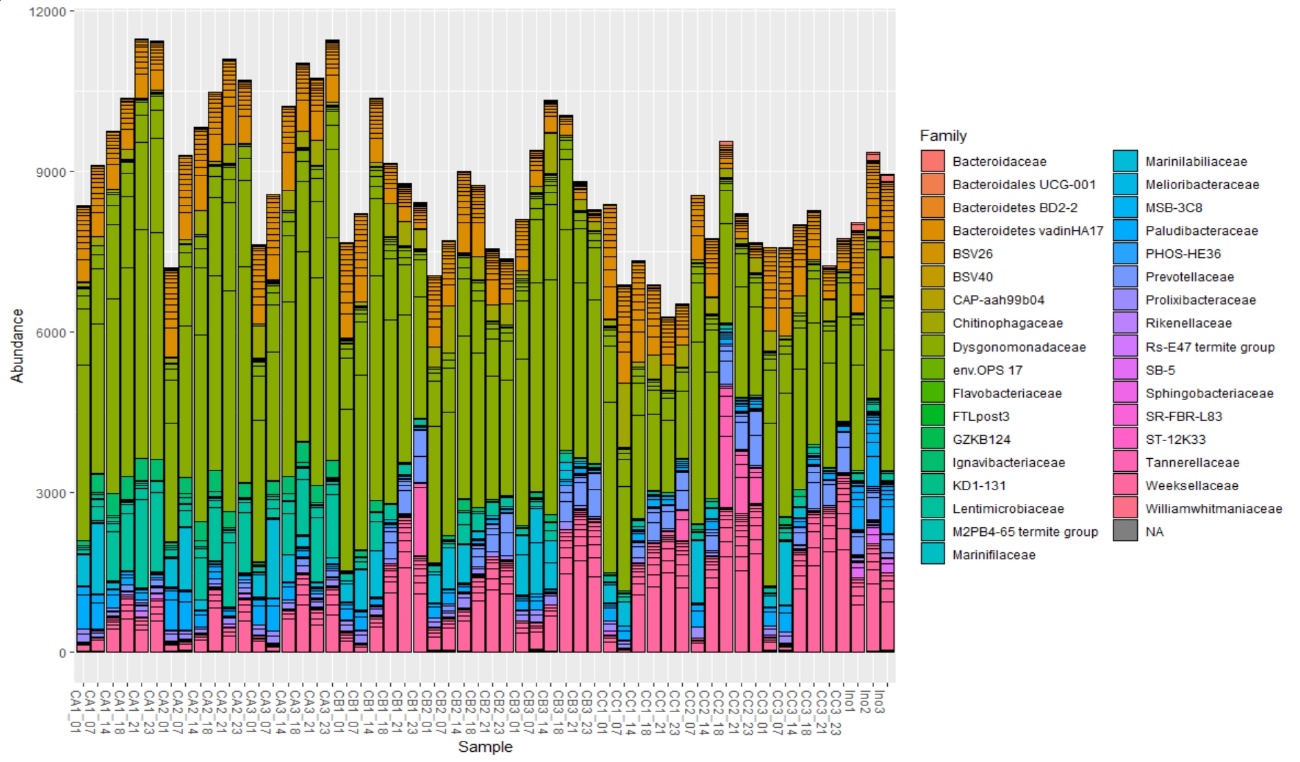


**Marinilabiliaceae**

**Melioribacteraceae**

**MSB-3C8**

**Paludibacteraceae**

**PHOS-HE36**

**Prevoteliaceae**

**Prolixibacteraceae**

**Rikeneliaceae**

**Rs-E47 termite group**

**SB-5**

**Sphingobacteriaceae**

**SR-FBR-L83**

**ST-12K33**

**Tannerellaceae**

**Weeksellaceae**

**Williamwhitmaniaceae**

**NA**


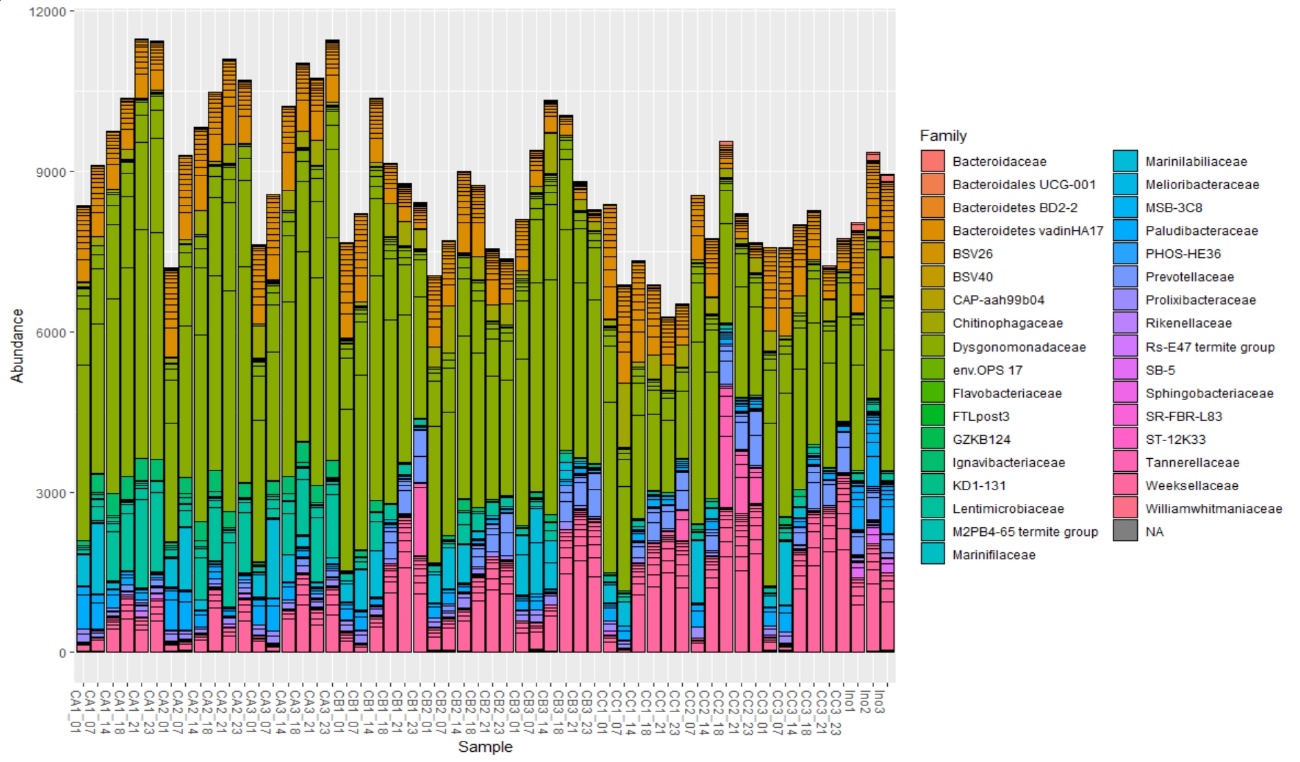


**Bacteroidales UCG-001**

**Bacteroidetes vadinHA17**

**BSV26**

**BSV40**

**Chitinophagaceae**

**env.OPS 17**

**FTLpost3**

**Ignavibacteriaceae**

**Lentimicrobiaceae**

**Bacteroidaceae**

**Bacteroidetes BD2-2**

**CAP-aah99b04**

**Dysgonomonadaceae**

**Flavobacteriaceae**

**GZKB124**

**KD1-131**

**M2PB4-65 termite group**

**Marinifilaceae**

Stacked bar plots display the relative abundance of Bacteroidota families in samples from control (CA), 0.6 bar H₂-supplemented (CB), and 0.9 bar H₂-supplemented (CC) anaerobic reactors, as well as the original inoculum (Ino). Assay CA: control (CA1–CA3 = triplicates 1–3); Assay CB: 0.6 bar H₂ (CB1–CB3 = triplicates 1–3); Assay CC: 0.9 bar H₂ (CC1–CC3 = triplicates 1–3). Day 1 (Adaptation 1), Day 7 (Adaptation 3), Day 14 (Cycle 3), Day 18 (Cycle 5), Day 21 (Cycle 6), and Day 23 (Cycle 7).

**Figure G:** Composition and abundance of *Chloroflexi* families across anaerobic fed-batch reactor samples.


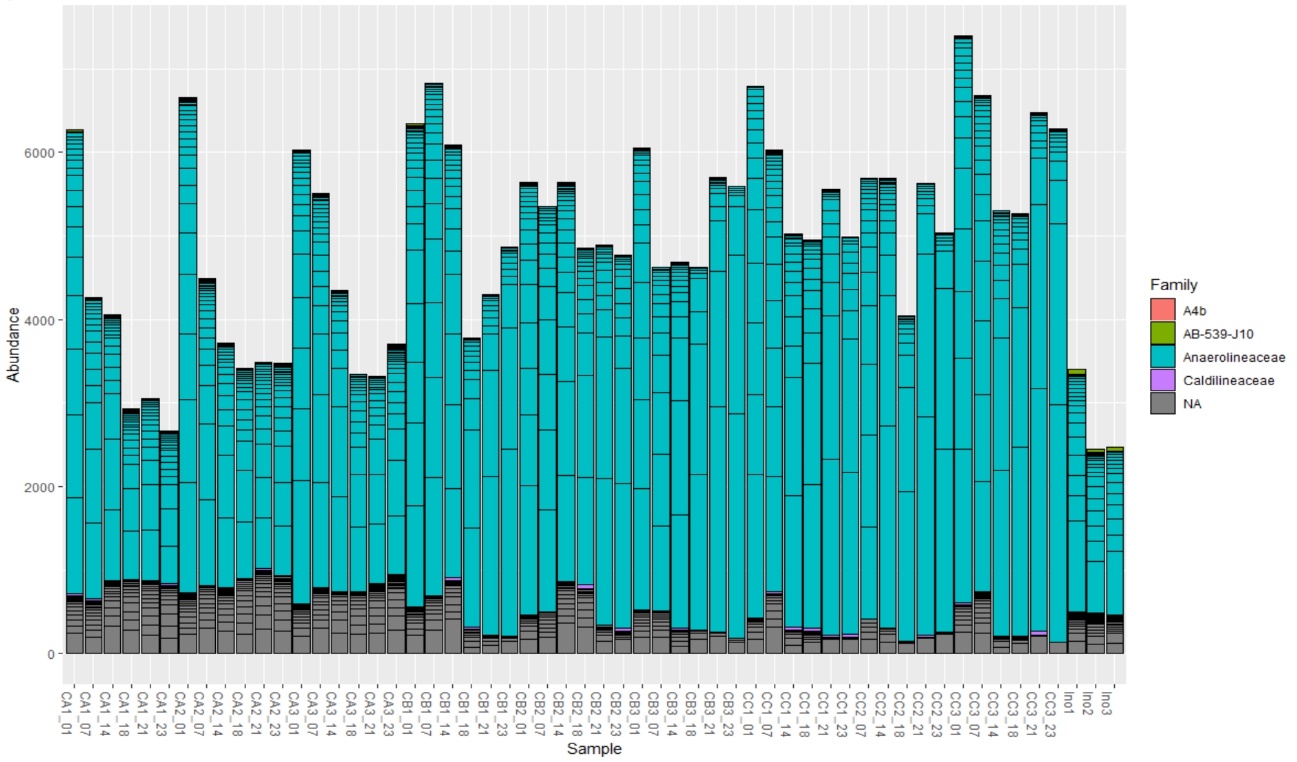


**CA1**

**CA2**

**CA3**

**CB1**

**CB2**

**CB3**

**CC1**

**CC2**

**CC3**

**Inoc**

**Abundance**

**Family**

**Sample**

**AB-539-J10**

**Caldilineaceae**

**A4b**

**Anaerolinaceae**

**NA**


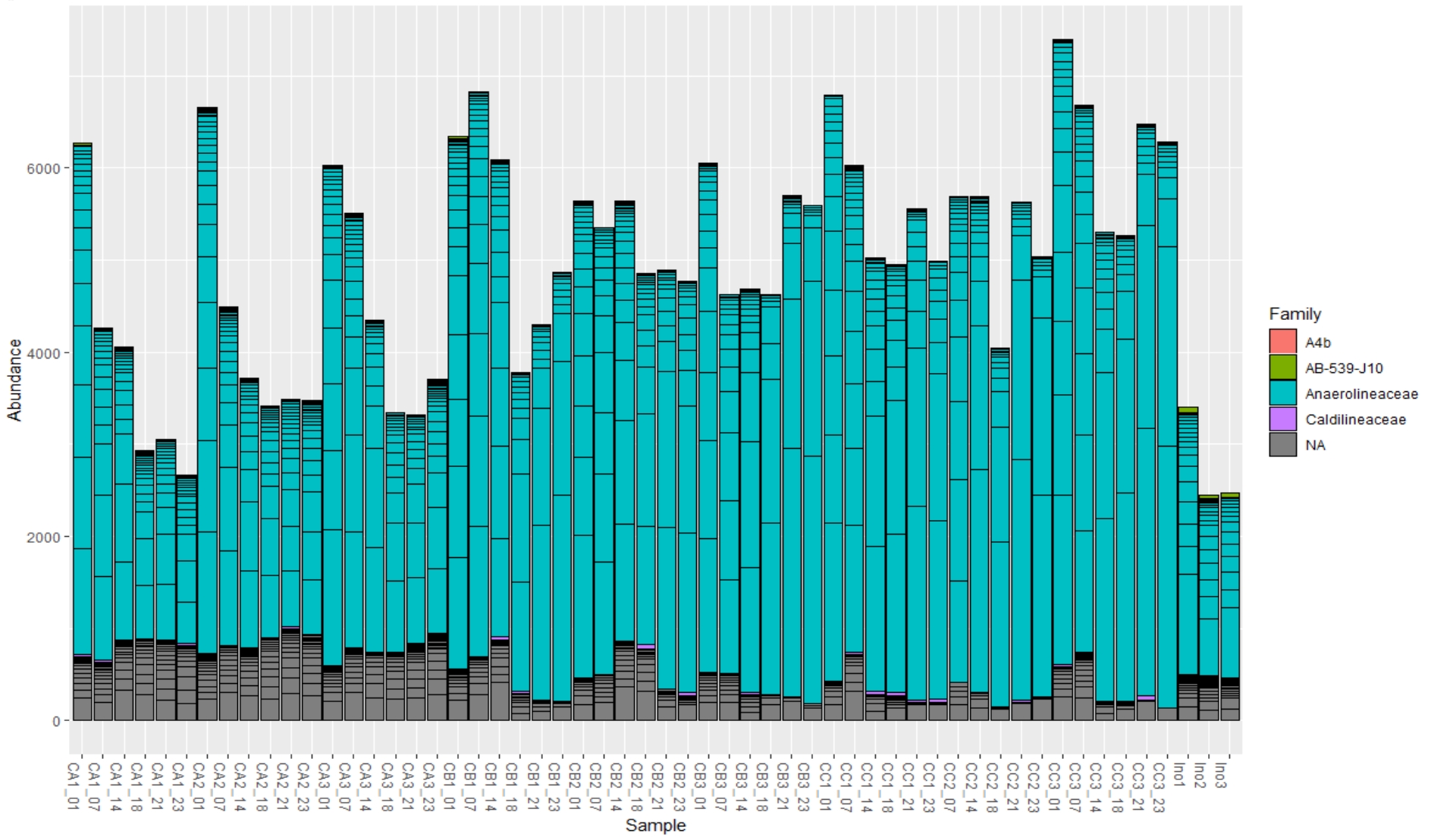


Stacked bar charts display the distribution of Chloroflexi families in control (CA), 0.6 bar H₂-supplemented (CB), and 0.9 bar H₂-supplemented (CC) reactors, as well as in the initial inoculum (Ino). The family Anaerolineaceae was dominant across nearly all samples, particularly under hydrogen-supplemented conditions, suggesting its role in syntrophic interactions under anaerobic conditions. Minor contributions were observed from Caldilineaceae, A4b, and AB-539-J10, whose presence may reflect niche metabolic adaptations or substrate preferences. Assay CA: control (CA1–CA3 = triplicates 1–3); Assay CB: 0.6 bar H₂ (CB1–CB3 = triplicates 1–3); Assay CC: 0.9 bar H₂ (CC1–CC3 = triplicates 1–3). Day 1 (Adaptation 1), Day 7 (Adaptation 3), Day 14 (Cycle 3), Day 18 (Cycle 5), Day 21 (Cycle 6), and Day 23 (Cycle 7).

**Figure J:** Relative abundance of *Firmicutes* families in anaerobic fed-batch reactors under different hydrogen partial pressures.

**Family**


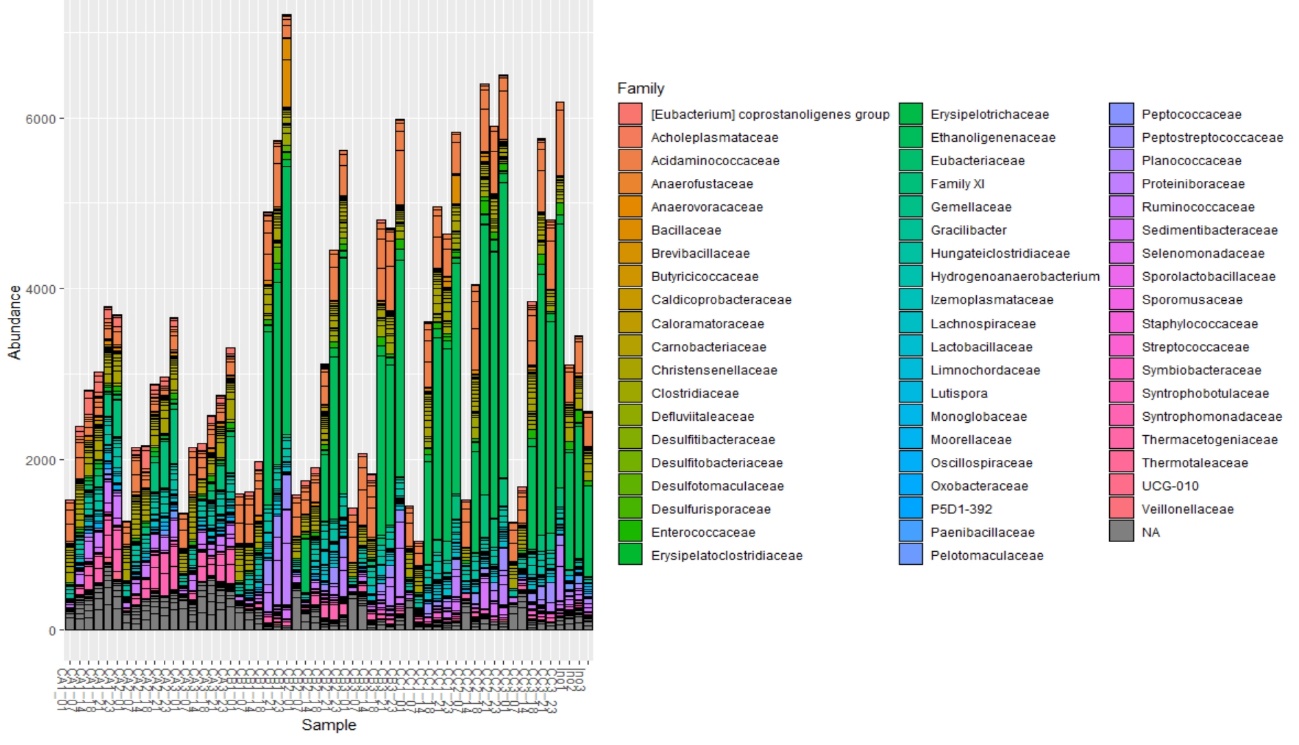


**[Eubacterium] coprostanoligenes group**

**Acholeplasmataceae**

**Acidaminococcaceae**

**Anaerofustaceae**

**Anaerovoracaceae**

**Bacillaceae**

**Brevibacillaceae**

**Butyricicoccaceae**

**Caldicoprobacteraceae**

**Caloramatoraceae**

**Carnobacteriaceae**

**Christensenellaceae**

**Clostridiaceae**

**Defluviitaleaceae**

**Desulfitibacteraceae**

**Desulfotomaculaceae**

**Desulfurisporaceae**

**Enterococcaceae**

**Erysipelatoclostridiaceae**

**Desulfitibacteriaceae**

**Erysipelotrichaceae**

**Ethanoligenenaceae**

**Eubacteriaceae**

**Family XI**

**Gemeliaceae**

**Gracilibacter**

**Hungateiclostridiaceae**

**Hydrogenoanaerobacterium**

**Izemoplasmataceae**

**Lachnospiraceae**

**Lactobacilaceae**

**Limnochordacea**

**Lutispora**

**Monoglobaceae**

**Moorellaceae**

**Oxobacteraceae**

**P5D1-392**

**Paenibacillaceae**

**Pelotomaculaceae**

**Oscillospiraceae**


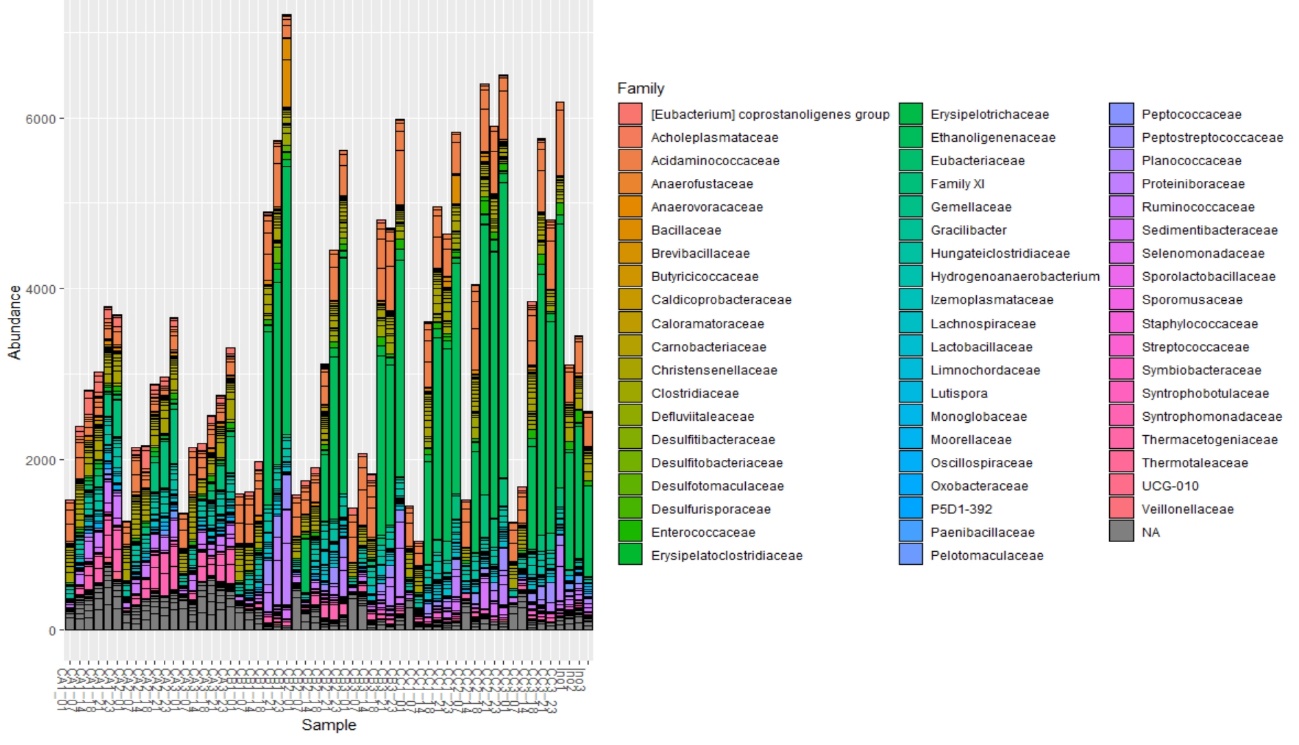


**Peptococcaceae**

**Peptostreptococcaceae**

**Planococcaceae**

**Proteiniboraceae**

**Ruminococcaceae**

**Sedimentibacteraceae**

**Selenomonadaceae**

**Sporolactobacillaceae**

**Sporomusaceae**

**Staphylococcaceae**

**Streptococcaceae**

**Symbiobacteraceae**

**Syntrophobotulaceae**

**Syntrophomonadaceae**

**Thermacetogeniaceae**

**UCG-010**

**Veilonellaceae**

**NA**

**Thermotaleaceae**


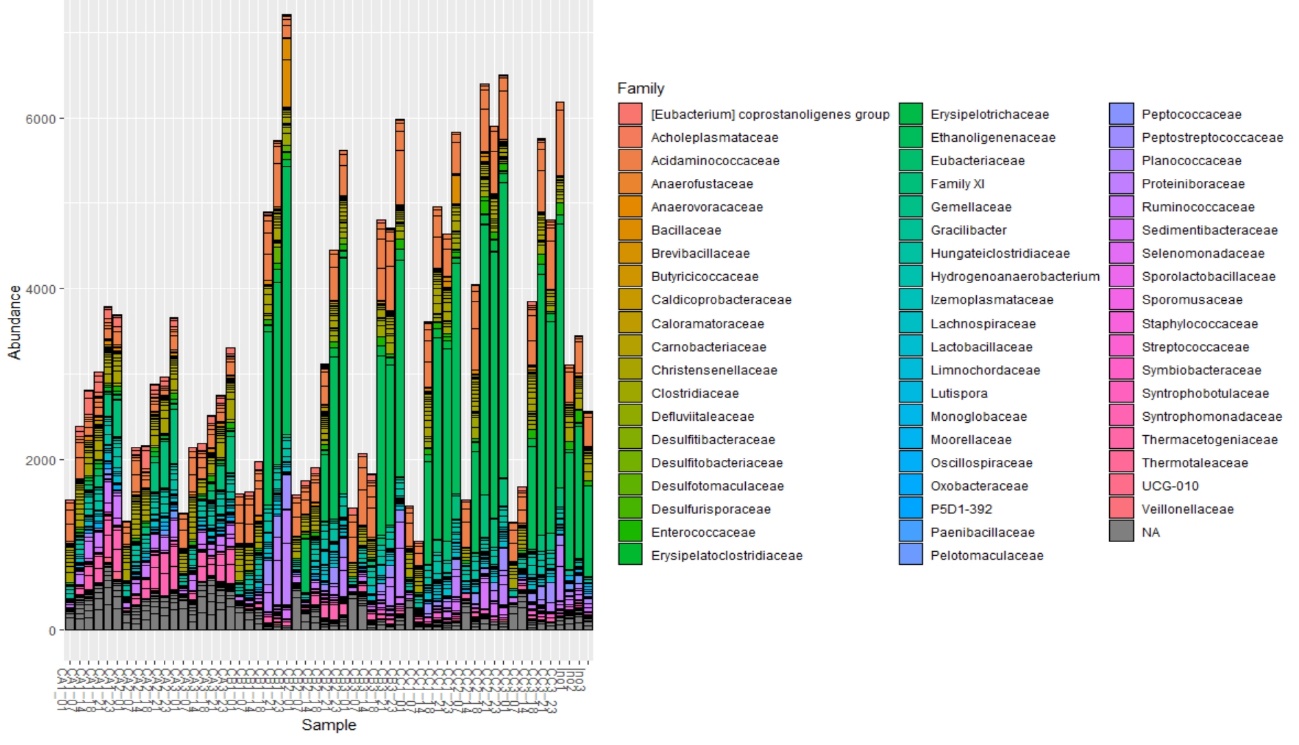


**CA1**

**CA2**

**CA3**

**CB1**

**CB2**

**CB3**

**CC1**

**CC2**

**CC3**

**Inoc**

**Abundance**

**Sample**


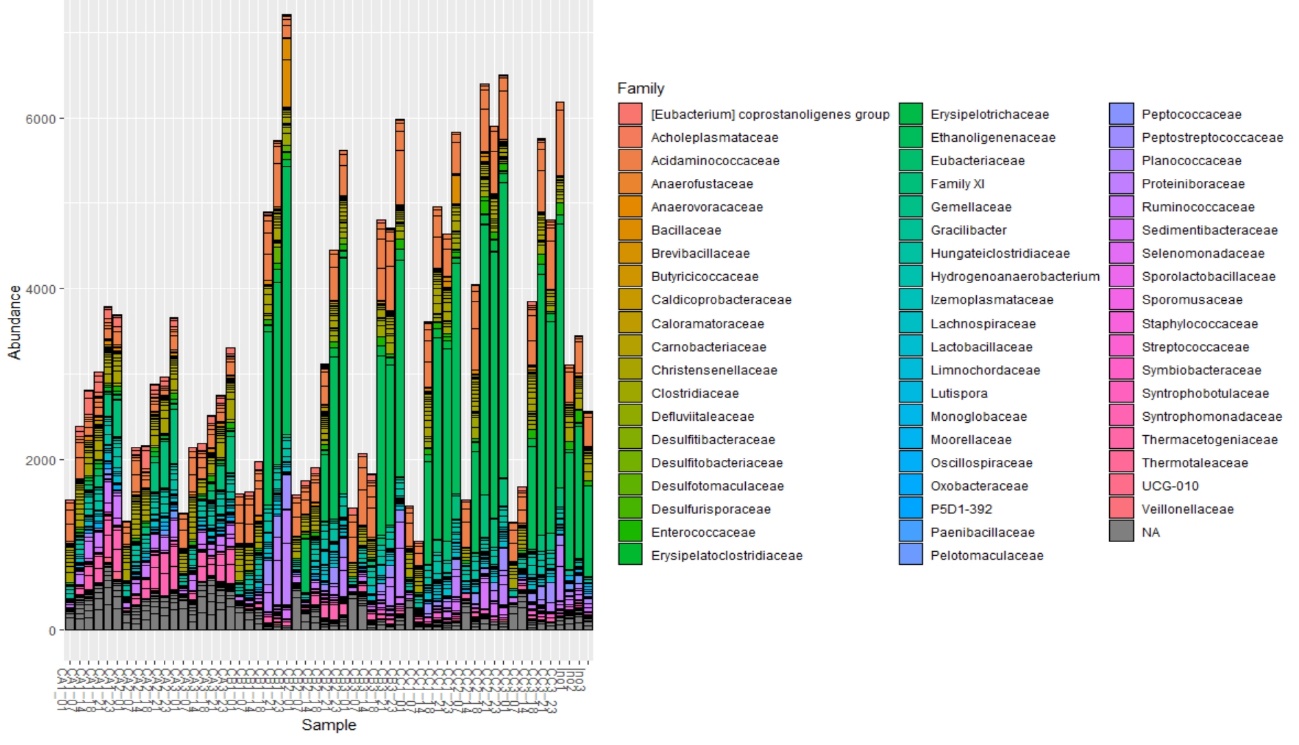


This stacked bar chart displays the family-level taxonomic composition of Firmicutes in control (CA), 0.6 bar H₂-supplemented (CB), and 0.9 bar H₂-supplemented (CC) samples, as well as the initial inoculum (Ino). Diverse fermentative and syntrophic families were detected, indicating a metabolically flexible Firmicutes community structure across the reactors. Assay CA: control (CA1–CA3 = triplicates 1–3); Assay CB: 0.6 bar H₂ (CB1–CB3 = triplicates 1–3); Assay CC: 0.9 bar H₂ (CC1–CC3 = triplicates 1–3). Day 1 (Adaptation 1), Day 7 (Adaptation 3), Day 14 (Cycle 3), Day 18 (Cycle 5), Day 21 (Cycle 6), and Day 23 (Cycle 7).
